# Understanding Microbial Community Dynamics in Up-Flow Bioreactors to Improve Mitigation Strategies for Oil Souring

**DOI:** 10.3389/fmicb.2020.585943

**Published:** 2020-12-03

**Authors:** Avishek Dutta, Ben Smith, Thomas Goldman, Leanne Walker, Matthew Streets, Bob Eden, Reinhard Dirmeier, Jeff S. Bowman

**Affiliations:** ^1^Integrative Oceanography Division, Scripps Institution of Oceanography, University of California San Diego, La Jolla, CA, United States; ^2^BP Upstream Technology, London, United Kingdom; ^3^BP Biosciences Center, San Diego, CA, United States; ^4^Rawwater Engineering Company Ltd., Culcheth, United Kingdom; ^5^Center for Microbiome Innovation, University of California, San Diego, La Jolla, CA, United States

**Keywords:** biosouring, mitigation, volatile fatty acids, microbial community, marine microbes

## Abstract

Oil souring occurs when H_2_S is generated in oil reservoirs. This not only leads to operational risks and health hazards but also increases the cost of refining crude oil. Sulfate-reducing microorganisms are considered to be the main source of the H_2_S that leads to oil souring. Substrate competition between nitrate-reducing and sulfate-reducing microorganisms makes biosouring mitigation via the addition of nitrate salts a viable strategy. This study explores the shift in microbial community across different phases of biosouring and mitigation. Anaerobic sand-filled columns wetted with seawater and/or oil were used to initiate the processes of sulfidogenesis, followed by mitigation with nitrate, rebound sulfidogenesis, and rebound control phases (via nitrate and low salinity treatment). Shifts in microbial community structure and function were observed across different phases of seawater and oil setups. Marine bacterial taxa (*Marinobacter*, *Marinobacterium*, *Thalassolituus*, *Alteromonas*, and *Cycloclasticus*) were found to be the initial responders to the application of nitrate during mitigation of sulfidogenesis in both seawater- and oil- wetted columns. Autotrophic groups (*Sulfurimonas* and *Desulfatibacillum*) were found to be higher in seawater-wetted columns compared to oil-wetted columns, suggesting the potential for autotrophic volatile fatty acid (VFA) production in oil-field aquifers when seawater is introduced. Results indicate that fermentative (such as Bacteroidetes) and oil-degrading bacteria (such as *Desulfobacula toluolica*) play an important role in generating electron donors in the system, which may sustain biosouring and nitrate reduction. Persistence of certain microorganisms (*Desulfobacula*) across different phases was observed, which may be due to a shift in metabolic lifestyle of the microorganisms across phases, or zonation based on nutrient availability in the columns. Overall results suggest mitigation strategies for biosouring can be improved by monitoring VFA concentrations and microbial community dynamics in the oil reservoirs during secondary recovery of oil.

## Introduction

Souring is a phenomenon in which the concentration of H_2_S increases in an oil reservoir. This increase in H_2_S leads to corrosion, poses a health risk to personnel involved in the production process, adversely affects downstream processes, and impacts the cost of oil production as well as the value of the produced oil itself (Gieg et al., 2011). Souring is a common occurrence in the petroleum industry, occurring both in terrestrial and offshore oil production operations. Souring generally takes place during secondary recovery when external fluids, mainly seawater, are injected to increase reservoir pressure and displace oil toward the production well. Injection of seawater introduces nutrients and marine microbiota to the oil reservoirs, greatly modifying the biogeochemical setting and composition of the *in situ* microbial community. A consequence of seawater injection is the introduction of significant amounts of sulfate, a major ion in seawater. Sulfate is used as an electron acceptor by a variety of sulfate-reducing microorganisms (SRMs), including sulfate-reducing bacteria (SRB) and archaea (SRA). H_2_S production is a direct outcome of the use of sulfate as a terminal electron acceptor. Although sulfidogenesis can be partly alleviated by discarding previously used water, recycling the water in combination with a mitigation strategy is economically and environmentally preferred.

Among different mitigation measures used to combat biosouring, nitrate injection in oil reservoirs has proven to be one of the most effective ways to mitigate biosouring (Gieg et al., 2011; Xue and Voordouw, 2015; Johnson et al., 2017; Prajapat et al., 2018; Wu et al., 2018). The addition of nitrate to the system enhances the growth of heterotrophic nitrate-reducing bacteria (hNRBs) and nitrate-reducing sulfur-oxidizing bacteria (NR-SOBs) (Gieg et al., 2011). The presence of hNRBs in the system lowers the pool of electron donors, such as VFAs and degraded hydrocarbons, likely because hNRBs outcompete SRBs for these resources. Additionally, NR-SOBs oxidize sulfide using nitrate as an electron donor. The outcome of this metabolism is nitrite, which has been observed to further inhibit sulfate reduction (Myhr et al., 2002; Gieg et al., 2011).

Previous *in situ* work suggests that pulsed injections of nitrate are more effective in suppressing sulfide production in comparison to continuous nitrate injection (Voordouw et al., 2009). Those authors postulated that SRMs in the near-well regions were replaced by hNRBs and NR-SOBs when nitrate was added, and continuous additions of nitrate made hNRBs dominant in the near-well regions (Voordouw et al., 2009). These hNRBs consumed most of the injected nitrate near the injection well, allowing sulfidogenesis to re-establish in the other parts of the reservoir. In contrast, a batch-wise increase in nitrate concentration can be used to break the zonation created by NRBs (near the injection well) and SRBs (further away from the injection well) (Voordouw et al., 2009).

Even though various effective and efficient mitigation strategies are well-studied (Gieg et al., 2011; Xue and Voordouw, 2015; Johnson et al., 2017; Prajapat et al., 2018; Wu et al., 2018), the microbial communities associated with different phases of biosouring and mitigation are relatively unexplored. Understanding microbial community structures and functions across different phases can significantly improve mitigation measures in oil fields. It is often hard to study these microbial community shifts in oil reservoirs due to their large volumes and challenges with *in situ* sampling. For this reason *in vitro* studies provide a valuable means to understand and compare the dynamics of microbial communities associated with biosouring and mitigation. Here 16S rRNA gene sequencing and metabolic inference were used to evaluate the shift in microbial community composition and metabolic functional potential when batch-wise nitrate was added to a soured system in the presence of seawater and oil.

## Materials and Methods

### Experimental Setup

**FIGURE 1:**
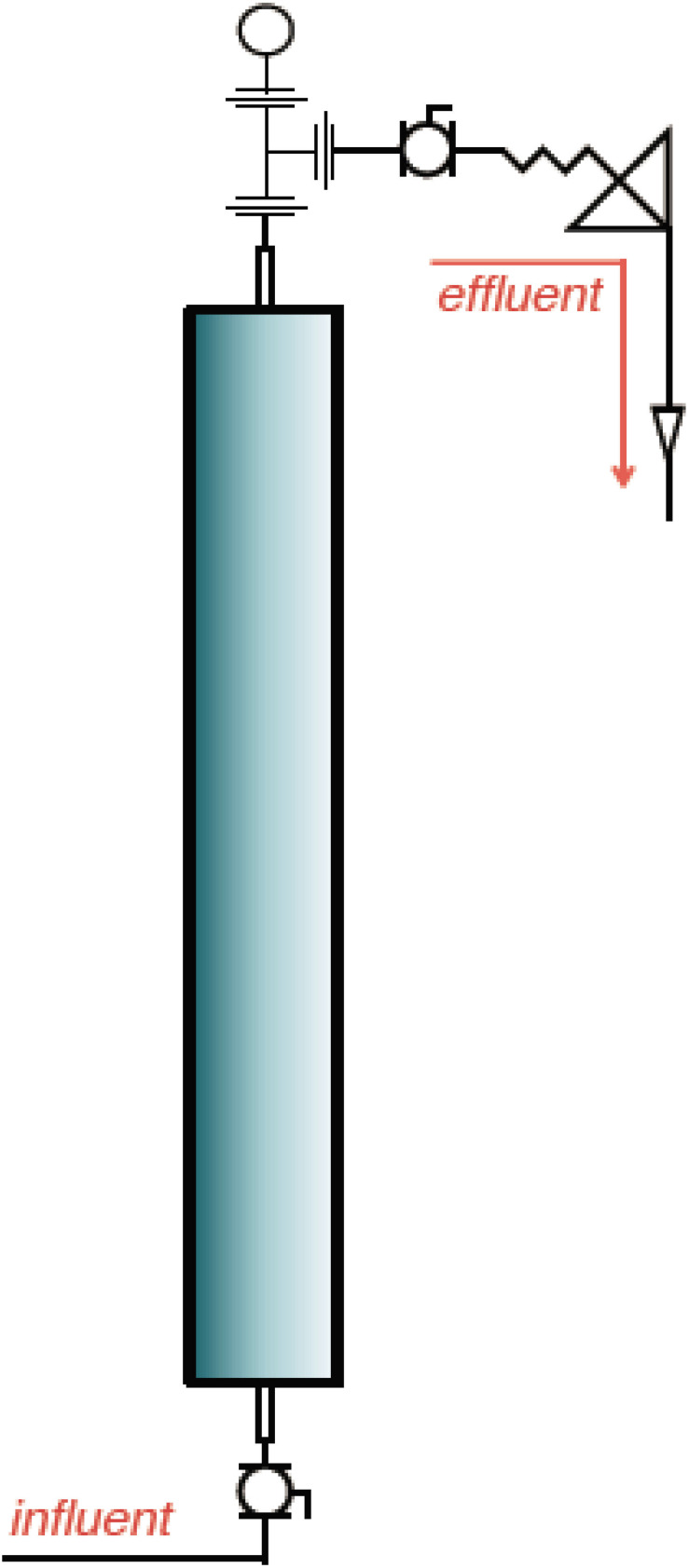
A representative schematic diagram of the bioreactors used in this study (Source: Rawwater Engineering Company Ltd.).

Six pressurized up-flow bioreactors (Figure 1) were used to understand the shift in microbial diversity across different phases of biosouring and mitigation. The bioreactors were packed with a mixture of ‘pre-soured’ 98% low-iron sand and 2% kaolin, by weight. Seawater was injected into all six bioreactors. Three of the columns were saturated with “dead” crude oil (crude oil that does not contain natural gas). The crude oil used in this study was classified as “medium crude oil” as per the American Petroleum Institute degree. The oil-wetted columns are referred to as OWCs, whereas seawater-wetted columns are referred to as SWCs. The bioreactors were operated under anaerobic conditions at 30°C and 18.27 MPa pressure to simulate the environment near the wellbore of an injector. This experimental setup was established at Rawwater Engineering Company Ltd.

Phases were designed to mimic the overall process of sulfidogenesis followed by nitrate control (M: application of nitrate for mitigation), rebound sulfidogenesis (RS: after stopping nitrate treatment), and rebound control (RC: mitigation of rebound sulfidogenesis). Sulfide concentrations were monitored across different timepoints in SWCs and OWCs, and M, RS, and RC phases were marked accordingly (Supplementary Table S1). Calcium nitrate salts were used in this study for mitigation. Rebound control phases were treated differently in SWCs and OWCs. In the seawater bioreactors, a low salinity (LS) treatment was used, whereas, in the OWCs, the re-application of nitrate was conducted. In the RC phase, the OWCs were chosen for re-application of nitrate because they were more relevant to the real-world scenario, and the influence of pulsed injection of nitrate on microbial community in oil fields would have been better represented in OWCs. Since low salinity flooding is emerging as a newer process, this mitigation strategy was implemented in SWCs to understand its influence on the microbial community. For LS treatment, two different sulfate concentrations were used in two different SWCs (10 mg L^–1^ in Column 2 and 100 mg L^–1^ in Column 3). VFAs were added in all the columns throughout the experiment in order to reduce the experimental variables between the different groups of pressurized bioreactors. Details of the experimental setup are given in the Supplementary Materials.

The effluent samples were collected to understand the microbial community dynamics within the near injection wellbore region (NIWR). Since the average residence time of the bioreactors was only 2.2 days, the effluent waters of the pressurized bioreactors represented the dynamic conditions within the NIWR. In the SWCs and OWCs, samples for microbial analyses were collected from three different phases. One sample each from three SWCs across M and RS phases, and one sample each from two SWCs in RC phase were used, whereas one sample each from three OWCs across M, RS, and RC phases were used for microbial analysis (Supplementary Table S1).

### DNA Extraction, Sequencing, and Bioinformatics Analyses

Sixty milliliter of effluent samples were filtered, and DNA was extracted from the filters using the Qiagen DNeasy Power Water Kit at Source Biosciences (United Kingdom), following the manufacturer protocol with an additional heating step to aid lysis. For a particular phase from each setup, the extracted DNA was pooled (Supplementary Table S2), vacuum concentrated, and eluted in 15 μL of molecular microbiology grade water. Bacterial 16S ribosomal RNA gene sequencing was performed using the Quick-16S NGS Library Preparation Kit (Zymo Research, Irvine, CA, United States). The proprietary bacterial 16S primers amplified the V3–V4 region of the 16S rRNA gene. The primers have been custom-designed by Zymo Research to provide the best coverage of the 16S rRNA gene while maintaining high sensitivity. Paired-end sequencing of the final library was conducted on the Illumina MiSeq platform with a v3 reagent kit (600 cycles). All library preparation and sequencing were performed at Zymo Research. Details of library preparation and sequencing are given in the Supplementary Materials. The sequence reads were submitted to the NCBI sequence read archive (SRA) under BioProject ID: PRJNA645907.

The reads generated from Illumina MiSeq were filtered, denoized, and merged using dada2 (Callahan et al., 2016). The final merged reads were analyzed using paprica v0.5.0 for the determination of community and predicted metabolic structure (Bowman and Ducklow, 2015^1^). Paprica was selected based on its explicit inclusion of genomes of relevant sulfur-reducing bacteria and past performance with nitrogen cycling genes (Arfken et al., 2017). In brief, paprica places each read on a phylogenetic reference tree created from complete 16S rRNA genes from all completed genomes in GenBank. Placements to terminal branches on the reference tree are referred to as closest completed genomes (CCG), while placements to internal branches are referred to as closest estimated genomes (CEG). The output of the paprica metabolic inference is an estimate of the enzymes and metabolic pathways contained in each member of the community. These data were further analyzed using custom Python scripts^2^. The taxonomic abundance file for all the edges (generated using downstream_paprica) was used to calculate alpha diversity indices using phyloseq (McMurdie and Holmes, 2013).

### Statistical Analysis

SIMPER analysis (based on Bray–Curtis dissimilatory) of the top five most abundant genera detected in each phase of biosouring and mitigation in both SWCs and OWCs were conducted using PAST3 (Hammer et al., 2001). Other statistical analyses were carried out in R and R Studio (Team RS, 2015). Welch’s two sample *t*-test was used to check whether alpha diversity indices were significantly different between the phases and to support the differential abundance of the different bacterial taxa. Sample S4 was not considered for the *t*-test of alpha diversity data since the value of Simpson’s index for S4 was found to be an outlier using Tukey’s method (Tukey, 1977). An observation was considered to be an outlier when its value was outside the range: [Q1 – 1.5 × (Q3 – Q1), Q3 + 1.5 × (Q3 – Q1)], where Q1 and Q3 are the first and third quartiles, respectively. DESeq2 (Love et al., 2014) was used to identify differentially present bacterial populations at different taxonomic levels across different phases in SWCs and OWCs. ANOSIM was used to compare the shift in microbial communities across different phases in SWCs and OWCs. Heatmap and cluster analyses were carried out with the pheatmap package (Kolde, 2019), and based on edges whose cumulative relative abundance was greater than 5%. Column clustering, which displayed the clustering of different samples, was based on Bray–Curtis dissimilatory. The clustsig package (Whitaker et al., 2014) in R was used to determine whether the column clusters were statistically significant. Each row (depicting relative percentage abundance of edges) was normalized using feature scaling and clustered based on correlation. For row clustering, pvclust (Suzuki and Shimodaira, 2006) was used to determine the significance of each cluster using approximately unbiased (AU) *p*-value using the bootstrap resampling technique.

Canonical analysis of principal coordinates (CAP) of Bray–Curtis dissimilatory based on the abundance of all edges across different samples was performed using phyloseq (McMurdie and Holmes, 2013) and vegan (Oksanen et al., 2007) where the genera most associated with the changes in community composition were analyzed. CAP analysis was also done based on enzyme composition across all the samples, as observed from the paprica results where relevant enzymes associated with the changes in the predicted enzymatic composition were plotted as vectors. Permutation tests for the significance of constraints in CAP analyses were done using the ANOVA function from the vegan package with 999 permutations. Before CAP analysis, edge abundance and enzyme abundance were converted to relative abundances using the transform_sample_counts function in phyloseq.

## Results and Discussion

This study reports the microbial community structure and function associated with different phases of souring and mitigation in SWCs and OWCs to improve the understanding of microbial community shifts. Exploring the microbial community dynamics across different phases in SWCs and OWCs will give us further insights in enhancing mitigation efficiencies and effectiveness.

### Comparison of Microbial Diversity Between SWCs and OWCs

Differences in the microbial community structure between SWCs and OWCs were evident from the comparative analyses. The average Shannon’s diversity index for SWCs (mean = 2.02, Standard deviation [SD] = 0.60) was more than for the OWCs (mean = 1.61, SD = 0.36) (Supplementary Figure S1 and Supplementary Table S3), which is statistically supported by Welch’s two sample *t*-test (*t* = 2.93, *p* = 0.012). Similar results were obtained for Simpson’s indices (Seawater: mean = 0.74, SD = 0.18 and Oil: mean = 0.68, SD = 0.10; *t* = 2.48. *p* = 0.026) (Supplementary Figure S1 and Supplementary Table S3). The differences in alpha diversity suggest that the presence of oil exerted strong selective pressure on the microbial community. This may be attributable to higher growth of specialist microorganisms in oil, or inhibition in oil caused by high concentrations of potentially antagonistic compounds. Microbial community diversity in the soured phase (in RS phases) was lower than the non-soured phases (M and RC phases) (Supplementary Figure S1). We propose two hypotheses for this. One, that H_2_S production inhibited the growth of other microorganisms present in the columns directly (Reis et al., 1992; O’Flaherty et al., 1998) or through the production of metal sulfides (Cole, 1977; Utgikar et al., 2002). Alternatively, that competition for the substrate suppressed the growth of non-dominant microbes. In that scenario, the proliferation of certain microbial populations (mainly SRBs) in the RS phase may have suppressed the growth of other microorganisms by utilizing the available electron donors and other nutrients present in the system (Abram and Nedwell, 1978; Dar et al., 2008).

Bacterial diversity was analyzed to understand the shift in community structure and function across different phases of mitigation and souring in SWCs and OWCs. Based on average relative abundance across all the samples, Proteobacteria appeared to be the most dominant phyla (mean = 93.34%, SD = 4.56%), followed by Bacteroidetes (mean = 4.21%, SD = 4.69%) (Supplementary Table S4). Bacteroidetes was seven-fold higher in OWCs compared to SWCs (Oil: mean = 7.02%, SD = 4.99%; Seawater: mean = 1.06%, SD = 0.46%; Oil vs. Seawater: *t* = 3.56, *p* = 0.007). This is consistent with previous studies where elevated Bacteroidetes were observed in hydrocarbon-contaminated sediments and oil-treated microcosms (Kim and Kwon, 2010; Koo et al., 2014, 2015; Atlas et al., 2015). Among the different phases in OWCs, percentage abundances of Bacteroidetes were found to be significantly higher in RS (mean = 11.45%, SD = 3.24%) and RC (mean = 8.60%, SD = 1.23%) phase while compared to M phase (mean = 1.01%, SD = 0.64%; RS vs. M: *t* = 5.47, *p* = 0.02; RC vs. M: *t* = 9.46, *p* = 0.002) (Supplementary Figure S2). Since Bacteroidetes are often characterized by their fermentative nature (Birg et al., 2019), their higher abundances in the RS and RC phases in OWCs suggests a potential for fermentation.

**FIGURE 2:**
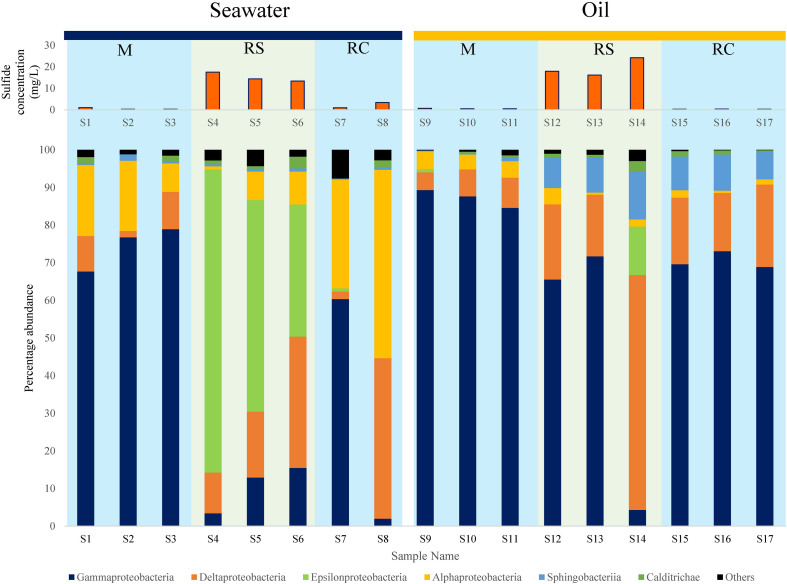
Distribution of different bacterial classes along with corresponding average sulfide generation across M (mitigation), RS (rebound sulfidogenesis), and RC (rebound control) phases in SWCs and OWCs. The blue boxes in the background represent non-sour phases, whereas the green boxes represent sour phases.

Class level diversity displayed a distinct shift in communities across different phases (Figure 2). In SWCs, Epsilonproteobacteria increased significantly in the RS phase compared to the M phase (Epsilonproteobacteria: RS: mean = 57.31%, SD = 22.75%; M: mean = 0%, SD = 0%) (Supplementary Table S5). In the SWCs and OWCs, Deltaproteobacteria were found to be higher in the RS compared to M phase (Deltaproteobacteria in SWCs: RS: mean = 21.11%, SD = 12.43%; M: mean = 7.01%, SD = 4.6%; Deltaproteobacteria in OWCs: RS: mean = 32.94%, SD = 25.65%; M: mean = 6.65%, SD = 1.71%) (Figure 2). In addition to Deltaproteobacteria, the elevated abundance of Epsilonproteobacteria in all the samples from the seawater RS phase suggests dynamic sulfur cycling since many of the members of Epsilonproteobacteria are known sulfur oxidizers (Ihara et al., 2017).

At the class level, phylum Bacteroidetes was mainly represented by Sphingobacteriia, whose shift in relative abundance followed the trend observed for Bacteroidetes (Figure 2 and Supplementary Table S6). The higher abundances of Sphingobacteriia in oil can be supported by the fact that many of the affiliated members of Sphingobacteriia can degrade oil, thus making them appropriate members of the bacterial community in OWCs (Szabó et al., 2011; Noparat et al., 2014; Chang et al., 2017). Moreover, a previous study has also reported the presence of Sphingobacteriia as a part of an oil-degrading bacterial community (Liu et al., 2017). The fermentative and oil-degrading nature of Bacteroidetes may indicate syntrophic behavior among SRBs/NRBs and Bacteroidetes, where the latter provides organic acids (VFAs) and/or H_2_ for the growth and sustenance of SRBs/NRBs (Zavarzin, 2010).

The lowest taxonomic affiliation assigned by paprica to all the edges was studied to get a better overview of the shift in community structure across different phases of biosouring and mitigation in SWCs and OWCs. During the M phase in SWCs, *Marinobacterium*, *Marinobacter*, *Alteromonas*, *Desulfobacula*, and *Cycloclasticus* were the five most dominant genera based on cumulative relative abundance (Supplementary Figure S3A and Supplementary Table S7). There was a drastic shift in the community structure in the seawater RS phase, where *Sulfurimonas*, *Desulfobacula*, *Marinobacterium*, *Desulfatibacillum*, and *Candidatus* Puniceispirillum were detected as the five most dominant genera. It was also interesting to note that during LS treatment in the seawater RC phase, *Pseudomonas*, *Desulfatibacillum*, *Hyphomonas*, *Candidatus* Puniceispirillum, and *Maricaulis* were found to be the most abundant genera. Most of the abundant genera observed across different phases of biosouring and mitigation are found in the marine environment (Abraham et al., 1999; Kasai et al., 2002; Badger et al., 2005; Lalucat et al., 2006; Oh et al., 2010; Callaghan et al., 2012; López-Pérez et al., 2012; Wöhlbrand et al., 2013; Han and Perner, 2015; Bae et al., 2018; Zhou et al., 2019). This suggests that even in the absence of an indigenous oil reservoir NRB and SRB population, microbial populations present in the injected seawater during secondary recovery have the capability to reduce sulfate and nitrate.

In SWCs, it was interesting to note that some of the closest relatives of the high abundance genera *viz*. *Marinobacterium aestuarii*, *Marinobacter* sp. LQ44, *Desulfobacula toluolica* Tol2, *Thalassolituus oleivorans* MIL-1, *Alcanivorax borkumensis* SK2, *Cycloclasticus* sp. P1, and *Desulfatibacillum alkenivorans* AK-01 observed across different phases have the capability to degrade aliphatic and/or aromatic hydrocarbons, making them suitable residents in the oil reservoirs (Kasai et al., 2002; Callaghan et al., 2012; Golyshin et al., 2013; Naether et al., 2013; Wöhlbrand et al., 2013; Bae et al., 2018; Zhou et al., 2019). These populations may also support biosouring, since previous studies reported sulfate reduction coupled with bacterial metabolism of mono-aromatic petroleum hydrocarbon (Pfiffner et al., 1997; Anderson and Lovley, 2000).

The cumulatively most abundant genera were also identified for the OWCs (Supplementary Figure S3B and Supplementary Table S8). When nitrate was applied during the oil M phase, *Marinobacterium*, *Thalassolituus*, *Marinobacter*, *Desulfobacula*, and *Alcanivorax* were found to be the most dominant bacterial genera. Bacterial community structure shifted during the oil RS phase, where *Marinobacterium*, *Desulfobacula*, *Sulfurimonas*, *Caldithrix*, and *Alteromonas* became the five most dominant bacterial genera. During the RC phase, a shift in the community structure was again observed where *Desulfobacula*, *Alcanivorax*, *Marinobacterium*, *Thalassolituus*, and *Pelobacter* became the most abundant bacterial genera. Though four of the five most abundant genera were common in the M and RC phases of OWCs, their percentage relative abundances varied between the phases. While comparing the oil RS phase with M and RC phases in OWCs, only two of the five most abundant genera were common. This indicates that the community structure of M and RC phases in OWCs were more similar when compared to the oil RS phase, suggesting that high sulfide concentrations or discontinuation of nitrate amendment are uniquely shaping the RS community or that the growth of some members carried over from the previous phase (M phase) is suppressed.

Comparing the shift in microbial community structure across different phases in SWCs and OWCs, it is evident that the shift is more significant in the SWCs compared to the OWCs. This suggests that the SWCs were more amenable to supporting diverse microbial communities. ANOSIM and SIMPER analysis was done to support the hypothesis. Results from ANOSIM suggested that microbial communities across different phases in SWCs varied more (*R* = 0.9728, *p* = 0.0042) while compared to the OWCs (*R* = 0.5802, *p* = 0.0045) which was further supported by SIMPER analysis which displayed that overall average dissimilarity across different phases in SWCs is more than that in OWCs (Supplementary Tables S9, S10). It should be noted that the shift in microbial diversity in the OWCs are more comparable to the real-world biosouring scenario while compared to the SWCs.

### Metabolic Potential of Bacterial Populations in M Phase

Distinct microbial communities were observed across souring and mitigation phases. Metabolic potentials of CCGs or CEGs of the most abundant genera observed across different phases in SWCs and OWCs were estimated to understand the community function in each phase. It was interesting to note that the most abundant CCGs and CEGs were differentially present across different phases of SWCs (Supplementary Table S11) and OWCs (Supplementary Table S12).

Nitrate salts were added to the system to achieve the M phase, where sulfidogenesis was suppressed. The metabolic potentials of the CCGs/CEGs in the M phase were determined to understand the community functions and the association of the most abundant bacterial populations with nitrate reduction. *Marinobacterium aestuarii* was the most abundant species observed in the M phase of SWCs and OWCs (Supplementary Tables S11, S12). The presence of *nrt* and *nirBD* in *M. aestuarii* suggests that they are capable of using extracellular nitrite and conducting nitrite reduction to ammonia (KEGG genome: T04398). It is possible that *M. aestuarii* uses an incomplete product of denitrification (i.e., nitrite) for their sustenance in the M phase. *Marinobacter* sp. LQ44 was one of the five most dominant CCGs observed in the M phase of OWCs and SWCs. They are not only capable of denitrification but possess the genetic capability to carry out dissimilatory nitrate reduction to ammonia (DNRA) (Zhou et al., 2019). *Cycloclasticus* sp. P1 (CCG of one of the five most abundant genera of the M phase for seawater) and *Thalassolituus oleivorans* MIL-1 (one of the five most abundant CCGs observed in the M phase of OWCs) also harbor *nrt* and *nirBD*, suggesting nitrite uptake and dissimilation (KEGG genome: T02265 and T04398 respectively). *Alcanivorax borkumensis* SK2, one of the abundant CCG observed in the M phase in OWCs, also has the potential for DNRA (KEGG genome: T00380).

A comparison of the metabolic potential of the most abundant microbial populations of the M phase suggests that DNRA is preferred over denitrification in the given system. Previous studies have shown that more reduced conditions and high C/N ratios favor DNRA over denitrification (Kraft et al., 2014; Kessler et al., 2018). The addition of VFAs in this system may have not only caused a reducing condition but also increased the C/N ratio, thus favoring DNRA over denitrification. Moreover, it has been previously observed that ATP synthesis in denitrification is far lower than expected from the free energy changes and even lower than in DNRA (Strohm et al., 2007). Thus nitrate/nitrite ammonifiers may achieve higher growth yields and outcompete the denitrifiers.

Interestingly, NR-SOBs, which play an essential role in mitigation (Gieg et al., 2011), were not observed among the abundant members of the bacterial communities of the M phase. There are two possible reasons for this observation. First, they were present at low abundance in the seawater/oil used in this study, hence were suppressed by the growth of other heterotrophic members. Second, the sulfide generated in the previous sulfidogenic phase escaped through the effluent leaving no trace of sulfide in the M phase, thus making it a substrate-limiting environment for the sulfur-oxidizing autotrophs.

### Metabolic Potential of Bacterial Populations in RS Phase

Rebound sulfidogenesis phase was achieved when the nitrate amendment in the system was stopped. Average sulfide concentrations across all the columns in the RS phase increased compared to the M phase (Supplementary Table S1). Metabolic potentials of the CCGs/CEGs in the RS phase were analyzed to determine the shift in overall community function and understand the association of the most abundant bacterial populations with sulfidogenesis. In the RS phase of SWCs and OWCs, the microbial diversity shifted where the relative abundance of the dominant members of the M phase (*Marinobacter* sp. LQ44, *T. oleivorans* MIL-1, *Alteromonas macleodii*, *Cycloclasticus* sp. P1) reduced significantly (Supplementary Table S13), and it seems that the discontinuation of nitrate addition in the system has deterred the growth of these nitrate-reducing bacterial populations. Though *D. toluolica* Tol2 was ubiquitously observed in all the phases, their relative abundance in each column increased in RS phase (compared to M phase) of SWCs and OWCs (Supplementary Figure S4). Another sulfate-reducing bacterial population, *Desulfatibacillum alkenivorans* AK-01, was also observed in the RS phase. Interestingly, a sulfur-oxidizing bacterium (SOB), *Sulfurimonas*, was found to be the most dominant taxon in the RS phase of SWCs (Supplementary Table S11). Co-occurrence of SRBs and SOBs were also previously observed in a water-flooded petroleum reservoir in China (Tian et al., 2017).

The presence of *Sulfurimonas* in higher abundance in RS phase (Supplementary Tables S11, S13) compared to other phases may be attributed to the presence of higher sulfide concentration. Moreover, *Sulfurimonas* does not necessarily compete with sulfate reducers for fixed carbon as they can fix carbon from inorganic sources (carbon dioxide and bicarbonate) (Han and Perner, 2015), making them suitable residents of the RS phase. However, sulfate reduction is favored in anoxic conditions, whereas sulfide oxidation is mostly favored when coupled with oxygen or nitrate reduction (Amend and Shock, 2001). Since these systems are anoxic, it is possible that nitrite, resulting from incomplete denitrification, may serve as electron acceptors for *Sulfurimonas* (Han and Perner, 2015).

**FIGURE 3:**
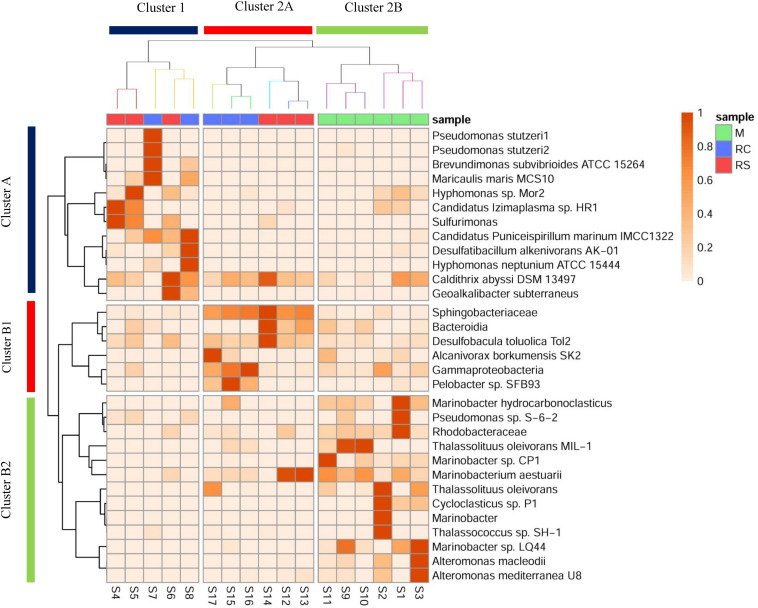
Heatmap showing a shift in the relative abundance of edges (whose cumulative percentage relative abundance is >5%) across different phases of biosouring and mitigation. Sample clustering was done based on Bray–Curtis dissimilarity, whereas taxon clustering was done based on correlation. Similar colored branches in the sample dendrogram indicate significant clusters (Supplementary Tables S16, S17). Significant clusters for taxon clustering are depicted in Supplementary Figure S6 and Supplementary Table S18.

It was interesting to note that even though similar conditions were maintained across the triplicate columns, microbial community structure varied across the triplicates for each phase. In the RS phase of OWCs, *Sulfurimonas* was only observed in sample S14, where the relative percentage abundance of Bacteroidetes was the highest (Figure 3 and Supplementary Figure S2). Since many *Sulfurimonas* species can use hydrogen as electron donor (Han and Perner, 2015), it seems that product of fermentation (i.e., hydrogen) from Bacteroidetes could have facilitated the growth of *Sulfurimonas* in sample S14. Also, this sample displayed the highest average sulfide generation compared to the other triplicate samples (Supplementary Table S1). Moreover, in the SWCs, the highest abundance of *Sulfurimonas* was observed in sample S4 (80.55%), where sulfide generation was highest (∼17.5 mg L^–1^) compared to other SWC setups. This further strengthens the previous hypothesis of sulfide as an instigator of *Sulfurimonas* growth. *Sulfurimonas* was found to be significantly higher in SWCs compared to OWCs (Supplementary Table S14). It seems that presence of oil aided the growth of hydrocarbon-degrading *D. toluolica* Tol2 (Wöhlbrand et al., 2013) in OWCs which suppressed the growth of *Sulfurimonas*.

### Metabolic Potential of Bacterial Populations in RC Phase

The RC phases were induced differently for SWCs and OWCs. LS treatment was applied to the SWCs, whereas nitrate doses were increased in the OWCs to achieve the RC phase. The differences in the treatment had a unique effect on shaping the microbial community structures. Metabolic potentials of the microbial community members in two distinct RC phases were analyzed to understand the differences in the impact of treatments on microbial community functions.

In the OWCs, nitrate dose was increased to suppress sulfidogenesis (Supplementary Table S1). The overall abundance of *Hyphomonas neptunium* ATCC 15444, *Maricaulis maris* MCS10, *T. oleivorans* MIL-1, and *Pseudomonas stutzeri* significantly increased in the RC phase compared to RS phase (Supplementary Table S15). *D. toluolica* Tol2, *A. borkumensis* SK2, *M. aestuarii*, and *T. oleivorans* MIL-1 were found to be the CCG of the most dominant taxa in oil RC phase (Supplementary Table S12). These taxa were also found to be the abundant members of the microbial communities of the oil M phase. This suggests that these bacteria, which were suppressed in the oil RS phase, again started proliferating in the RC phase.

Low salinity treatment was applied to the seawater columns to mitigate sulfidogenesis and initiate the RC phase. Two different sulfate concentrations were used in two different columns, one with slightly higher sulfate (S8) than the other (S7). In sample S7, *Pseudomonas stutzeri* was observed to be the most abundant taxon followed by *Ca.* Puniceispirillum marinum and *Maricaulis maris* (Supplementary Table S11). The diazotrophic nature of *P. stutzeri* (Lalucat et al., 2006) supports its presence in higher abundance in S7 (Supplementary Table S11). *P. stutzeri* may be fixing nitrogen in the low salinity sample, thus increasing the bioavailable nitrogen pool in the system to sustain the growth of other bacterial populations. *D. alkenivorans* AK-01 was observed to be the CCG of the most abundant taxon in S8, followed by *H. neptunium* ATCC 15444 and *Ca.* Puniceispirillum marinum (Supplementary Table S11). The abundance of *D. alkenivorans* AK-01 was ∼30 times higher in S8 (37.7%) compared to S7 (1.29%), which corroborates the higher influent sulfate and effluent sulfide concentration in S8 while compared to S7 (Supplementary Table S1). It was also interesting to note that the most of the abundant genera observed in S7 and S8 are generalist in nature (Abraham et al., 1999; Badger et al., 2005; Lalucat et al., 2006; Oh et al., 2010; Callaghan et al., 2012). These taxa were either absent in other samples or were present in very low abundances (Supplementary Tables S11, S12), which suggests that lower salinity has helped these bacterial groups to thrive while suppressing the growth of other bacteria with a higher salinity preference. There is another possibility that high nitrate and sulfate concentrations in other phases have selected for specialists whose higher growth and nutrient utilization rate have suppressed the growth of these generalists.

Moreover, LS treatment triggered certain microbial members in the SWCs which used lesser amount of VFAs compared to microbial communities in other phases. The effluent VFAs were higher in the RC phase of SWCs (compared to other phases, SWC-RC: mean = 25.93 mg L^–1^, SD = 3.52 mg L^–1^; Rest of the samples: mean = 0.62 mg L^–1^, SD = 0.60 mg L^–1^; *t* = 10.12, *p* = 0.062), suggesting a lack of VFA utilization and/or increased VFA production in the system. It seems that the microbial community is self-sustaining and does not depend on the external VFA inputs. The higher abundance of chemolithotrophic SRB, *D. alkenivorans* AK-01 (Kuever, 2014) in the seawater RC phase while compared to the presence of chemoorganotrophic SRB, *D. toluolica* Tol2 (Kuever, 2014) in other phases (Supplementary Tables S11, S12) further supports the previous hypothesis. Lower utilization of VFAs may also be attributed to the reduced abundance of electron acceptors (mainly sulfate) in the LS treatment while compared to the other phases. Overall results suggest that chemoorganotrophic processes are lower during LS treatment compared to the other phases.

In addition to the marked differences observed in the microbial community structure of oil and seawater RC phases, sulfide generation also varied across these two setups. While compared to the oil RC phase (average sulfide production = 0.34 mg mL^–1^, SD = 0.007 mg mL^–1^) (Supplementary Figure S5), higher sulfide concentrations were detected in both the seawater RC samples (sulfide concentration: S7 = 1 mg mL^–1^ and S8 = 3.4 mg mL^–1^), even though influent sulfate concentration was notably higher for the oil RC phase compared to seawater RC phase (Supplementary Table S1). It seems that adding nitrate has more impact on suppressing sulfidogenesis compared to the LS treatment.

### Comparison of Taxonomy and Metabolic Potential of Microbial Populations Across Different Phases

The presence of different microbial populations with varied metabolic potentials across different phases in OWCs and SWCs suggested syntrophic interactions among various community members. A heatmap with clustering was constructed to gain additional insight into the shift in microbial communities and to understand the probable interactions among different community members (Figure 3). Eight significant clusters (*p* < 0.05) based on Bray–Curtis dissimilatory were obtained for the sample clustering (Supplementary Tables S16, S17), which can be grouped into two broad clusters (Cluster 1 and Cluster 2). In Cluster 1, the samples from the RC phase of seawater (S7 and S8) clustered with S6 (sample from RS phase of seawater). Clustering of S6 and S8 suggests that there might be some carryover of microbial populations from the sulfidogenic phase when LS treatment was applied since both the samples are from the same column (Supplementary Table S1). This supports the higher production of H_2_S in LS based mitigation while compared to nitrate amendment (Supplementary Figure S5). Cluster 2 contains two distinct sub-clusters (Clusters 2A and 2B). Cluster 2A showed two distinct clusters of samples from the RC and RS phase of OWCs. This suggests that there is more similarity in the microbial diversity among the intra-phasic samples while compared to the inter-phasic samples from the RC and RS phase of OWCs. Cluster 2B displayed the grouping of samples from the M phase of SWCs and OWCs. This indicates that in the M phase, the presence of oil does not have a significant effect on selecting the most abundant community members. From the overall sample clustering in the heatmap, it can be concluded that the microbial community structure in the RS phases may have a role in shaping the RC communities.

The clustering of the most abundant taxa was based on the correlation matrix, and the significance of each cluster was determined using approximately unbiased (AU) *p*-value (Supplementary Figure S6 and Supplementary Table S18) using a bootstrap resampling technique. Clusters were considered significant for AU > 95, indicating 95% probability of not being a random cluster. Similar to the grouping of samples, two broad clusters for the taxon clustering were observed (Cluster A and Cluster B, AU > 95). Cluster A consisted of taxa whose abundance was found to be highest in samples present in Cluster 1, whereas Cluster B1 and Cluster B2 included taxa whose abundance was found to be highest in samples from Cluster 2A and 2B, respectively. This suggests that the samples were clustered based on a set of taxa whose abundance was found to be highest in that sample cluster. Most of the taxa present in Cluster A are either metabolic generalists (*P. stutzeri*, *M. maris, H. neptunium, Candidatus* Puniceispirillum marinum) (Abraham et al., 1999; Badger et al., 2005; Lalucat et al., 2006; Oh et al., 2010) and/or carbon-fixers (such as *Sulfurimonas*, *D. alkenivorans* AK-01, and *Caldithrix abyssi*) (Miroshnichenko et al., 2003; Callaghan et al., 2012; Han and Perner, 2015). This suggests that there might be a small guild of organisms where some specialist carbon fixers such as sulfur-oxidizing *Sulfurimonas* and sulfate-reducing *D. alkenivorans* AK-01 are fixing carbon, which is further being utilized by the generalists/heterotrophs.

The close associations of *D. toluolica*, *Bacteroidia*, and *Sphingobacteriaceae* in the Cluster B1 suggest that the organic acids produced by *Bacteroidia* and *Sphingobacteriaceae* either by fermentation or carbohydrate oxidation (Lambiase, 2014; Birg et al., 2019) are used by *D. toluolica* as an electron donor for sulfate reduction. Though VFAs are added in the system, the co-occurrence of these bacterial groups suggests that organic acids might be the limiting substrate in the process of chemoorganotrophic sulfate reduction. This is further supported by the presence of chemolithotrophic groups (*Sulfurimonas* and *D. alkenivorans*) in higher abundance in SWCs while compared to OWCs (Supplementary Tables S11, S12, S14). Since organotrophic SRBs are presumed to utilize the VFAs, the pool of labile carbon may have been reduced in the SWCs, creating a niche for autotrophs. On the contrary, even if the VFA pool in the OWCs is utilized, fermentative organisms and oil-degraders can replenish it.

Most of the taxa present in Cluster B2 (*Marinobacter*, *Marinobacterium*, *Thalassolituus*, *Alteromonas*, and *Cycloclasticus*) are found in the marine environment (as discussed above). Interestingly, these groups were found to be in higher abundances in samples from the M phase of SWCs and OWCs. As noted earlier, many of these bacterial taxa are known nitrate/nitrite reducers, giving the impression that the native seawater communities are the initial responders to the application of nitrate during mitigation.

**FIGURE 4:**
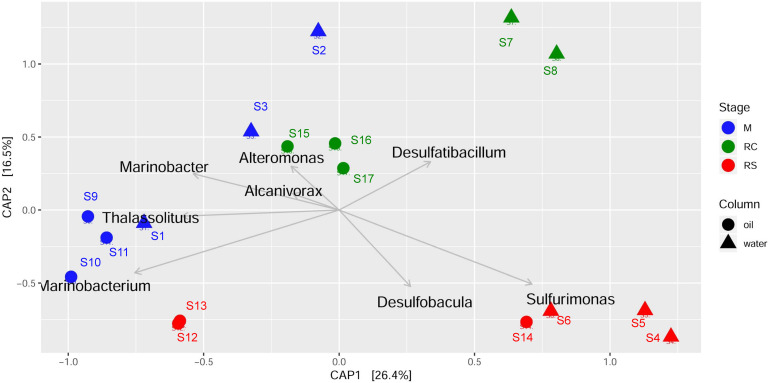
Canonical analysis of principal coordinates (CAP) of Bray–Curtis dissimilatory matrix based on relative abundance of all edges observed across different phases of biosouring and mitigation.

Canonical analysis of principal coordinates analyses were done to determine the grouping of different samples based on community structures and functions. Associations of different bacterial genera/enzymes with different samples were further validated using CAP analyses. CAP analysis based on relative abundance of all the edges across different samples (Figure 4) supported the previous clustering, as shown in the heatmap (Figure 3). Associations of different abundant genera with the samples from different phases were also observed, which further supports the results observed from clustering analysis. ANOVA shows that these associations are statistically significant (*F* = 4.23, *p* = 0.001). Though each of the phases in different column types clustered separately, the samples from the M phase and RC phase were closely clustered except for the samples from the seawater RC phase, which suggests that LS treatment has a distinct effect on the microbial community while compared to nitrate treatment.

**FIGURE 5:**
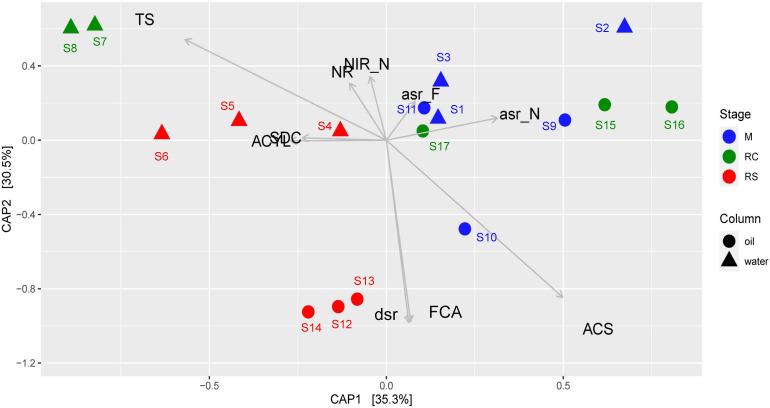
Canonical analysis of principal coordinates (CAP) of Bray–Curtis dissimilatory matrix based on relative abundance of all enzymes observed across different phases of biosouring and mitigation. Abbreviations of enzyme names as used in the CAP analysis are as follows: asr_F, assimilatory sulfite reductase (ferredoxin); asr_F, assimilatory sulfite reductase (NADPH); ACYL, ATP citrate synthase; ACS, acetyl-CoA synthetase; dsr, dissimilatory sulfite reductase; FCA, formate *C*-acetyltransferase; NR, nitrate reductase; NIR_N, nitrate reductase (NADH); SDC, sulfite dehydrogenase; TS, thiosulfate sulfurtransferase.

Canonical analysis of principal coordinates analysis based on relative abundances of different enzymes across different phases of biosouring and mitigation also yielded similar results (Figure 5), where the associations of the relevant enzyme with the samples are statistically significant (ANOVA, *F* = 8.828, *p* = 0.001). Assimilatory sulfite reductases (*asr*) were found to be in higher abundances in the non-sour (M and RC) phases compared to the sour phases of SWCs and OWCs, whereas dissimilatory sulfite reductase (*dsr*) was found to be in higher abundance in the oil RS phase. This suggests that the microorganisms in the non-sour phases are assimilating sulfate (present in seawater) while in the sour phases, sulfate is mainly used for anaerobic respiration. CAP analysis also suggested a higher presence of acetyl-CoA synthetase (*acs*) and formate C-acetyltransferase (*pfl*D) in OWCs compared to SWCs. The higher presence of *dsr* along with *acs* and *pfl*D in the OWCs further strengthens the previous hypothesis of chemoorganotrophic sulfate reduction and possible interactions among fermentative microorganisms and sulfate reducers. On the other hand, the association of sulfite dehydrogenase and ATP citrate synthase in the seawater RS phase suggests that sulfur oxidation and autotrophy are prevalent in this phase, which is in line with the taxonomy data. Comparable trends obtained from both CAP analyses indicated that the taxonomy of different bacterial groups across different phases reflects the bacterial community function.

### Persistence of Certain Microbial Populations Across Different Phases

The persistence of certain microbial populations in all the phases was investigated to gain insight into their adaptation abilities. On analyzing the shift in most abundant taxa across different phases in SWCs and OWCs, there appear to be some bacterial genera carried over from one phase to the other. It seems that bacteria may be phenotypically plastic to be competitive in the geochemical environment reflected in the new phase. Zonation caused due to contrasting substrate availability in different sections of the columns may also lead to the persistence of certain microbial populations. In an up-flow bioreactor, the influent substrates may be utilized by the bottom populations, creating a dearth of nutrients in the upper section, which may trigger a very different population. *Desulfobacula toluolica* was one of the most abundant taxa, which was observed across all the samples (Supplementary Tables S11, S12). Though this SRB is not known to possess the capability to reduce nitrate, their presence in the M and RC phases indicate versatile metabolic capabilities. Their growth can be sustained by the sulfate from the influent seawater, but the absence of sulfide production indicates a shift in metabolic lifestyle or dormancy of *D. toluolica*. Previous work suggests that SRBs can shift to fermentative lifestyle from sulfidogenic lifestyle (Plugge et al., 2011). It seems that *D. toluolica* is adapting to fermentation in the M and RC phases, but the fermentative nature of *D. toluolica* is not reported in any previous literature.

## Conclusion

This study investigated the shift in microbial community structure and function across different phases of biosouring and mitigation in the presence of seawater and oil. The microbial community was more diverse in SWCs compared to OWCs. Distinct microbial communities with different metabolic potentials were observed across different phases of biosouring and mitigation. Microbial communities responded differently in the presence and absence of oil. Autotrophic groups were found to be higher in abundance in SWCs compared to OWCs, suggesting the potential for autotrophic VFA production in oil-field aquifers. Persistence of certain microbial populations across different phases may be due to the shift in metabolic lifestyle of microbes across phases, or zonation based on nutrient availability in the up-flow bioreactors. Results indicate that fermentative and oil-degrading bacteria play an important role in generating electron donors in the system, which sustains biosouring and nitrate reduction. Overall results suggest mitigation strategies can be improved by monitoring VFA concentration and microbial diversity in the reservoir, which can affect sulfidogenesis, nitrate-reducing pathway selection, and abundances of heterotrophic and autotrophic communities.

## Data Availability Statement

The sequencing data can be found under the BioProject ID PRJNA645907.

## Author Contributions

AD carried out the bioinformatics analyses, statistical analyses, and data organization. AD and JB contributed conception and developed the first draft of the manuscript. BS, LW, MS, and BE designed and conducted the experiments. TG, RD, and JB assisted in data interpretation and manuscript preparation. JB conceived the study and done overall mentoring. All authors contributed to the article and approved the submitted version.

## Conflict of Interest

BS was employed by BP. TG and RD were employed by BP Biosciences Center. LW, MS, and BE were employed by Rawwater Engineering Company Ltd. The remaining authors declare that the research was conducted in the absence of any commercial or financial relationships that could be construed as a potential conflict of interest.

## Footnotes

1 https://github.com/bowmanjeffs/paprica/releases/tag/paprica_v0.5.0

2 https://github.com/avishekdutta14/downstream_paprica
