# Supplementary material for: Understanding Microbial Community Dynamics in Up-Flow Bioreactors to Improve Mitigation Strategies for Oil Souring
Source: Front Microbiol. 2020 Dec 3;11:585943. doi: 10.3389/fmicb.2020.585943 (PMC7744764; doi:10.3389/fmicb.2020.585943)
Supplement: Supplementary file 1 [file Data_Sheet_1.docx]

**Supporting information**

**Understanding microbial community dynamics in up-flow bioreactors to improve mitigation strategies for oil souring**

Avishek Dutta^1*^, Ben Smith^2^, Thomas Goldman^3^, Leanne Walker^4^, Matthew Streets^4^, Bob Eden^4^, Reinhard Dirmeier^3^, Jeff S Bowman^1,5^

^1^Integrative Oceanography Division, Scripps Institution of Oceanography, UC San Diego

^2^BP Upstream Technology

^3^BP Biosciences Center

^4^Rawwater Engineering Company Ltd.

^5^Center for Microbiome Innovation, UC San Diego

**Corresponding author:**

Avishek Dutta

Email ID: avdutta@ucsd.edu; avishekdutta14@gmail.com

**Materials and Methods**

**Experimental Setup**

Six pressurized up-flow bioreactors were used to understand the shift in microbial diversity across different phases of biosouring and mitigation. The bioreactors were packed with a mixture of ‘pre-soured’ 98% low-iron sand, and 2% Kaolin by weight. The pre-soured sand used in this study consisted of core matrix material from decommissioned, pressurized bioreactors that operated in a previous bioreactor study. The core material was extracted from the ‘Control,’ and ‘Nitrate-dosed’ bioreactor groups from the previous study, and the columns were known to contain a mature, pre-established oilfield microbiological consortium. Packing with ‘pre-soured’ sand significantly reduces the time to observe inferred microbiological activity from chemical analysis (e.g., sulfide production, nitrate reduction, VFA utilization), hence no prolonged period at the start of the new bioreactor study for an initial establishment phase.

Seawater was injected in all the six bioreactors. Three of the bioreactors were seawater-wetted, and the other three were oil-wetted. Seawater used in this study is composed of 90% synthetic seawater (Instant Ocean) and 10% Irish Sea seawater mix. The natural seawater used in the pressurized bioreactor contains an average of 2,800 mg/L sulfate, < 0.1 mg/L nitrate, and < 0.1mg/L VFA. The pH of the natural seawater typically varied between 8.0 and 8.5. The detailed composition of natural and synthetic seawater is present in Table S19. In the oil-wetted bioreactor, columns were saturated with dead crude oil. The bioreactors were operated at 30 ℃ and 18.27 MPa. Anaerobiosis was maintained in the influent vessels by a positive pressure N_2_ gas head. Oxygen scavenger (potassium metabisulfite) was also added to the influent if dissolved O_2_ concentrations were greater than 0.5 mg L^-1^. A continuous injection method was used to inject influent at a target flow rate of 0.05 mL min^-1^.

Phases were designed to mimic the overall process of sulfidogenesis followed by nitrate control (application of nitrate for mitigation) [M], rebound sulfidogenesis (after stopping nitrate treatment) [RS], and rebound control (mitigation of rebound sulfidogenesis) [RC]. Shifts between phases in seawater- and oil- wetted columns were marked by shifts in average sulfide concentrations (Supporting Table S1). Rebound control phases were treated differently in seawater- and oil- wetted columns. In the seawater bioreactors, a low salinity (LS) treatment was used, whereas, in the oil-wetted columns, the re-application of nitrate was conducted for the RC phase. In the LS treatment, seawater was replaced with deionized water having sulfate salt in it. Two different concentrations of the salt were used in two different columns (10 mg L^-1^ in sample S7 and 100 mg L^-1^ in S8). VFAs were added in all the columns throughout the experiment. VFAs consisted of acetate (33 mg L^-1^), formate (3.3 mg L^-1^), propionate (1.65 mg L^-1^), and butyrate (1.65 mg L^-1^). Calcium nitrate salts were used in this study for mitigation. The concentrations of nitrate ions used in different phases are mentioned in Supporting Table S1.

In the seawater- and oil- wetted columns, samples for microbial analyses were collected from three different phases (M, RS, and RC). Seventeen samples were analyzed for this study. Three samples each from the M and RS phases and two samples from the RC phase were used for seawater columns, whereas for oil columns, three samples each from M, RS, and RC phases were used.

**Library preparation and sequencing**

Bacterial 16S ribosomal RNA gene-targeted sequencing was performed using the Quick-16S NGS Library Preparation Kit (Zymo Research, Irvine, CA). These proprietary bacterial 16S primers amplified the V3-V4 region of the 16S rRNA gene. The primers have been custom-designed by Zymo Research to provide the best coverage of the 16S gene while maintaining high sensitivity. The sequencing library was prepared using an innovative library preparation process in which PCR reactions were performed in real-time PCR machines to control cycles and therefore prevent PCR chimera formation. The final PCR products were quantified with qPCR fluorescence readings and pooled together based on equal molarity. The final pooled library was cleaned up with Select-a-Size DNA Clean & Concentrator (Zymo Research, Irvine, CA), then quantified with TapeStation (Agilent) and Qubit (Life Technologies). The final library was sequenced on the Illumina MiSeq with a v3 reagent kit (600 cycles). The sequencing was performed with >10% PhiX spike-in. All library preparation and sequencing were performed at Zymo Research.

**Tables**

**Table S1:** Nutrients in effluents and influents of different bioreactors across M (mitigation), RS (rebound sulfidogenesis), and RC (rebound control) phases of seawater and oil enrichments

| Sample  Name | Column  type | Phases | Column | Effluent Average  Sulfide  (mg L^-1^) | Effluent Average  VFA  (mg L^-1^) | Influent  Average  Sulfate (mg L^-1^) | Influent  Nitrate  (mM) |
| --- | --- | --- | --- | --- | --- | --- | --- |
|  |  |  |  |  |  |  |  |
| S1 | Seawater | M | Column 1 | 1.1 | 0.4 | 2241.15 | 0.54 |
| S2 | Seawater | M | Column 2 | 0.3 | 0.4 | 2240.72 | 0.54 |
| S3 | Seawater | M | Column 3 | 0.3 | 0.4 | 2240.23 | 0.54 |
| S4 | Seawater | RS | Column 1 | 17.5 | 0.2 | 1762.45 | 0 |
| S5 | Seawater | RS | Column 2 | 14.4 | 0.2 | 1867.73 | 0 |
| S6 | Seawater | RS | Column 3 | 13.4 | 0.2 | 1880.82 | 0 |
| S7 | Seawater | RC | Column 2 | 1.0 | 28.4 | 12.54 | 0 |
| S8 | Seawater | RC | Column 3 | 3.4 | 23.4 | 103.92 | 0 |
| S9 | Oil | M | Column 4 | 0.7 | 0.5 | 2241.98 | 1.07 |
| S10 | Oil | M | Column 5 | 0.5 | 0.4 | 2241.98 | 1.07 |
| S11 | Oil | M | Column 6 | 0.5 | 0.4 | 2241.98 | 1.07 |
| S12 | Oil | RS | Column 4 | 17.9 | 0.4 | 2146.67 | 0.54 |
| S13 | Oil | RS | Column 5 | 16.2 | 0.4 | 2146.67 | 0.54 |
| S14 | Oil | RS | Column 6 | 24.2 | 0.4 | 2155.39 | 0.54 |
| S15 | Oil | RC | Column 4 | 0.3 | 2.2 | 2239.03 | 1.54 |
| S16 | Oil | RC | Column 5 | 0.4 | 1.9 | 2239.03 | 1.54 |
| S17 | Oil | RC | Column 6 | 0.3 | 1.0 | 2239.03 | 1.54 |

**Table S2:** Details of pooled samples for DNA extraction from different columns across different phases

| **Sample Name** | **Column name** | **Extraction Date** | **Sample Name** | **Column name** | **Extraction Date** |
| --- | --- | --- | --- | --- | --- |
| S1 | Column 1 | 19-05-2016 | S9 | Column 4 | 19-05-2016 |
|  | Column 1 | 02-06-2016 |  | Column 4 | 02-06-2016 |
|  | Column 1 | 09-06-2016 |  | Column 4 | 09-06-2016 |
|  | Column 1 | 15-06-2016 | S10 | Column 5 | 19-05-2016 |
|  | Column 1 | 23-06-2016 |  | Column 5 | 02-06-2016 |
| S2 | Column 2 | 19-05-2016 |  | Column 5 | 09-06-2016 |
|  | Column 2 | 02-06-2016 | S11 | Column 6 | 19-05-2016 |
|  | Column 2 | 09-06-2016 |  | Column 6 | 02-06-2016 |
|  | Column 2 | 15-06-2016 |  | Column 6 | 09-06-2016 |
|  | Column 2 | 23-06-2016 | S12 | Column 4 | 20-07-2016 |
| S3 | Column 3 | 19-05-2016 |  | Column 4 | 27-07-2016 |
|  | Column 3 | 02-06-2016 |  | Column 4 | 04-08-2016 |
|  | Column 3 | 09-06-2016 |  | Column 4 | 10-08-2016 |
|  | Column 3 | 15-06-2016 |  | Column 4 | 18-08-2016 |
|  | Column 3 | 23-06-2016 | S13 | Column 5 | 20-07-2016 |
| S4 | Column 1 | 23-08-2016 |  | Column 5 | 27-07-2016 |
|  | Column 1 | 30-08-2016 |  | Column 5 | 03-08-2016 |
|  | Column 1 | 07-09-2016 |  | Column 5 | 10-08-2016 |
|  | Column 1 | 14-09-2016 |  | Column 5 | 18-08-2016 |
|  | Column 1 | 21-09-2016 | S14 | Column 6 | 20-07-2016 |
| S5 | Column 2 | 23-08-2016 |  | Column 6 | 27-07-2016 |
|  | Column 2 | 30-08-2016 |  | Column 6 | 03-08-2016 |
|  | Column 2 | 07-09-2016 |  | Column 6 | 10-08-2016 |
|  | Column 2 | 14-09-2016 |  | Column 6 | 18-08-2016 |
|  | Column 2 | 21-09-2016 | S15 | Column 4 | 21-09-2016 |
| S6 | Column 3 | 23-08-2016 |  | Column 4 | 28-09-2016 |
|  | Column 3 | 30-08-2016 |  | Column 4 | 05-10-2016 |
|  | Column 3 | 07-09-2016 |  | Column 4 | 19-10-2016 |
|  | Column 3 | 14-09-2016 |  | Column 4 | 26-10-2016 |
|  | Column 3 | 21-09-2016 | S16 | Column 5 | 21-09-2016 |
| S7 | Column 2 | 25-10-2016 |  | Column 5 | 28-09-2016 |
|  | Column 2 | 02-11-2016 |  | Column 5 | 05-10-2016 |
|  | Column 2 | 08-11-2016 |  | Column 5 | 19-10-2016 |
|  | Column 2 | 16-11-2016 |  | Column 5 | 26-10-2016 |
|  | Column 2 | 23-11-2016 | S17 | Column 6 | 21-09-2016 |
| S8 | Column 3 | 26-10-2016 |  | Column 6 | 28-09-2016 |
|  | Column 3 | 02-11-2016 |  | Column 6 | 05-10-2016 |
|  | Column 3 | 08-11-2016 |  | Column 6 | 19-10-2016 |
|  | Column 3 | 16-11-2016 |  | Column 6 | 26-10-2016 |
|  | Column 3 | 23-11-2016 |  |  |  |

**Table S3:** Sequence information and alpha diversity indices

|  | **Column  type** | **Phases** | **input** | **filtered** | **denoisedF** | **denoisedR** | **merged** | **Observed  edges** | **Shannon** | **Simpson** | **InvSimpson** | **Fisher** |
| --- | --- | --- | --- | --- | --- | --- | --- | --- | --- | --- | --- | --- |
| **S1** | Seawater | M | 122104 | 121666 | 117465 | 110090 | 107439 | 44 | 2.40 | 0.85 | 6.72 | 4.35 |
| **S2** | Seawater | M | 103519 | 103114 | 98355 | 94737 | 91424 | 48 | 2.61 | 0.87 | 7.62 | 4.88 |
| **S3** | Seawater | M | 124071 | 123579 | 119772 | 109686 | 107008 | 55 | 2.66 | 0.89 | 9.39 | 5.58 |
| **S4** | Seawater | RS | 138886 | 138368 | 133169 | 129081 | 125929 | 21 | 0.88 | 0.34 | 1.52 | 1.89 |
| **S5** | Seawater | RS | 126137 | 125673 | 119702 | 109618 | 105624 | 18 | 1.49 | 0.64 | 2.75 | 1.62 |
| **S6** | Seawater | RS | 67108 | 66887 | 59114 | 56881 | 51420 | 18 | 2.05 | 0.81 | 5.17 | 1.75 |
| **S7** | Seawater | RC | 104245 | 103906 | 99817 | 93904 | 90789 | 43 | 2.18 | 0.75 | 4.08 | 4.32 |
| **S8** | Seawater | RC | 82927 | 82623 | 74095 | 73866 | 67326 | 22 | 1.92 | 0.77 | 4.29 | 2.12 |
| **S9** | Oil | M | 111024 | 110632 | 107594 | 101288 | 99647 | 29 | 1.90 | 0.78 | 4.45 | 2.76 |
| **S10** | Oil | M | 87711 | 87367 | 83608 | 78430 | 76296 | 23 | 1.46 | 0.68 | 3.09 | 2.20 |
| **S11** | Oil | M | 124475 | 123987 | 120540 | 111877 | 109870 | 39 | 2.23 | 0.79 | 4.87 | 3.80 |
| **S12** | Oil | RS | 135304 | 134827 | 130976 | 124677 | 122699 | 21 | 1.27 | 0.58 | 2.36 | 1.90 |
| **S13** | Oil | RS | 114729 | 114319 | 110647 | 103044 | 101071 | 17 | 1.19 | 0.54 | 2.17 | 1.53 |
| **S14** | Oil | RS | 94430 | 94080 | 90162 | 84283 | 82292 | 13 | 1.26 | 0.57 | 2.34 | 1.16 |
| **S15** | Oil | RC | 94331 | 93996 | 91282 | 86896 | 85010 | 35 | 1.94 | 0.76 | 4.19 | 3.46 |
| **S16** | Oil | RC | 85636 | 85318 | 82413 | 77030 | 74730 | 26 | 1.45 | 0.63 | 2.67 | 2.53 |
| **S17** | Oil | RC | 103254 | 102906 | 100322 | 94134 | 91896 | 20 | 1.75 | 0.78 | 4.64 | 1.85 |

**Table S4:** Relative percentage abundance of most abundant phylum in SWCs and OWCs

|  | S1 | S2 | S3 | S4 | S5 | S6 | S7 | S8 | S9 | S10 | S11 | S12 | S13 | S14 | S15 | S16 | S17 | Average | Standard Deviation |
| --- | --- | --- | --- | --- | --- | --- | --- | --- | --- | --- | --- | --- | --- | --- | --- | --- | --- | --- | --- |
| Proteobacteria | 96.01 | 97.11 | 96.44 | 95.67 | 94.25 | 94.25 | 92.17 | 94.71 | 99.61 | 98.76 | 97.03 | 89.86 | 88.68 | 81.52 | 89.28 | 89.19 | 92.20 | 93.34 | 4.56 |
| Bacteroidetes | 0.82 | 1.73 | 0.88 | 0.54 | 1.30 | 0.86 | 1.69 | 0.64 | 0.39 | 0.96 | 1.67 | 8.79 | 10.49 | 15.06 | 9.00 | 9.59 | 7.22 | 4.21 | 4.69 |
| Calditrichaeota | 1.85 | 0.00 | 1.39 | 1.00 | 0.65 | 3.11 | 0.00 | 1.89 | 0.00 | 0.28 | 0.55 | 0.98 | 0.83 | 2.79 | 1.41 | 1.07 | 0.46 | 1.08 | 0.92 |
| Tenericutes | 0.48 | 0.62 | 0.00 | 2.36 | 1.61 | 0.00 | 0.00 | 0.00 | 0.00 | 0.00 | 0.00 | 0.23 | 0.00 | 0.00 | 0.00 | 0.00 | 0.00 | 0.31 | 0.67 |
| Ignavibacteriae | 0.00 | 0.00 | 0.00 | 0.00 | 0.63 | 0.00 | 2.33 | 1.81 | 0.00 | 0.00 | 0.00 | 0.00 | 0.00 | 0.00 | 0.00 | 0.00 | 0.00 | 0.28 | 0.70 |
| Chloroflexi | 0.00 | 0.37 | 0.00 | 0.00 | 0.39 | 0.00 | 1.26 | 0.63 | 0.00 | 0.00 | 0.76 | 0.14 | 0.00 | 0.63 | 0.00 | 0.00 | 0.00 | 0.25 | 0.37 |
| Acidobacteria | 0.00 | 0.00 | 0.79 | 0.30 | 1.05 | 1.09 | 0.00 | 0.00 | 0.00 | 0.00 | 0.00 | 0.00 | 0.00 | 0.00 | 0.00 | 0.00 | 0.00 | 0.19 | 0.39 |
| Firmicutes | 0.63 | 0.00 | 0.00 | 0.00 | 0.12 | 0.00 | 1.10 | 0.00 | 0.00 | 0.00 | 0.00 | 0.00 | 0.00 | 0.00 | 0.31 | 0.00 | 0.12 | 0.13 | 0.30 |
| Spirochaetes | 0.22 | 0.17 | 0.49 | 0.12 | 0.00 | 0.69 | 0.00 | 0.32 | 0.00 | 0.00 | 0.00 | 0.00 | 0.00 | 0.00 | 0.00 | 0.15 | 0.00 | 0.13 | 0.20 |
| Actinobacteria | 0.00 | 0.00 | 0.00 | 0.00 | 0.00 | 0.00 | 1.46 | 0.00 | 0.00 | 0.00 | 0.00 | 0.00 | 0.00 | 0.00 | 0.00 | 0.00 | 0.00 | 0.09 | 0.35 |
| Unassigned | 0.00 | 0.00 | 0.00 | 0.00 | 0.00 | 0.00 | 0.00 | 0.00 | 0.00 | 0.00 | 0.00 | 0.00 | 0.00 | 0.00 | 0.00 | 0.00 | 0.00 | 0.00 | 0.00 |

**Table S5:** DESeq2 results showing significant fold changes of bacterial classes between RS vs. M phases of seawater-wetted columns (SWCs)

|  | log2FoldChange | lfcSE | stat | *p*-value | *p-*adj |
| --- | --- | --- | --- | --- | --- |
| Epsilonproteobacteria | 18.73 | 1.36 | 13.72 | 7.22E-43 | 5.77E-42 |
| Gammaproteobacteria | -2.70 | 0.61 | -4.45 | 8.61E-06 | 3.45E-05 |

**Table S6:** DESeq2 results showing significant fold changes of bacterial classes between RS vs. M phases of oil-wetted columns (OWCs)

|  | log2FoldChange | lfcSE | stat | *p*-value | *p*-adj |
| --- | --- | --- | --- | --- | --- |
| Sphingobacteriia | 2.82 | 0.38 | 7.45 | 9.62E-14 | 8.66E-13 |
| Alphaproteobacteria | -2.59 | 0.91 | -2.86 | 0.00423 | 0.01904 |

|  | S1 | S2 | S3 | Mean | SD | S4 | S5 | S6 | Mean | SD | S7 | S8 | Mean | SD |
| --- | --- | --- | --- | --- | --- | --- | --- | --- | --- | --- | --- | --- | --- | --- |
| Phase | M | M | M | M | M | RS | RS | RS | RS | RS | RC | RC | RC | RC |
| *Marinobacterium* | 30.44 | 5.54 | 11.52 | 15.83 | 12.99 | 0.00 | 0.00 | 10.03 | 3.34 | 5.79 | 1.23 | 0.00 | 0.62 | 0.87 |
| *Marinobacter* | 14.50 | 9.69 | 21.73 | 15.30 | 6.06 | 0.00 | 0.00 | 0.00 | 0.00 | 0.00 | 0.00 | 0.00 | 0.00 | 0.00 |
| *Alteromonas* | 0.26 | 13.32 | 26.37 | 13.32 | 13.06 | 1.12 | 0.00 | 3.38 | 1.50 | 1.72 | 0.00 | 0.00 | 0.00 | 0.00 |
| *Desulfobacula* | 6.21 | 1.71 | 7.49 | 5.13 | 3.04 | 8.52 | 17.54 | 21.27 | 15.78 | 6.56 | 0.74 | 2.46 | 1.60 | 1.21 |
| *Cycloclasticus* | 2.32 | 8.94 | 2.69 | 4.65 | 3.72 | 0.00 | 0.00 | 0.00 | 0.00 | 0.00 | 0.00 | 0.00 | 0.00 | 0.00 |
| *Pseudomonas* | 8.99 | 0.03 | 0.00 | 3.01 | 5.18 | 0.81 | 1.27 | 0.00 | 0.69 | 0.64 | 58.78 | 1.96 | 30.37 | 40.17 |
| *Desulfatibacillum* | 2.62 | 0.00 | 1.37 | 1.33 | 1.31 | 2.34 | 0.00 | 6.89 | 3.08 | 3.50 | 1.29 | 37.70 | 19.50 | 25.75 |
| *Hyphomonas* | 0.89 | 0.78 | 1.03 | 0.90 | 0.13 | 0.51 | 3.18 | 1.14 | 1.61 | 1.40 | 4.13 | 26.79 | 15.46 | 16.02 |
| *Ca.* Puniceispirillum | 0.00 | 0.00 | 0.75 | 0.25 | 0.43 | 0.00 | 3.17 | 4.52 | 2.56 | 2.32 | 8.19 | 12.49 | 10.34 | 3.04 |
| *Sulfurimonas* | 0.00 | 0.00 | 0.00 | 0.00 | 0.00 | 80.55 | 56.27 | 35.09 | 57.31 | 22.75 | 0.79 | 0.00 | 0.39 | 0.56 |
| *Maricaulis* | 0.00 | 0.00 | 0.00 | 0.00 | 0.00 | 0.00 | 1.18 | 0.00 | 0.39 | 0.68 | 5.77 | 2.87 | 4.32 | 2.05 |

**Table S7:** Average relative percentage abundance of most abundant genera in SWCs with the mean and standard deviation of M, RS, and RC phases

|  | S9 | | S10 | | S11 | | Mean | | SD | | S12 | | S13 | | S14 | | Mean | | SD | | S15 | | S16 | | S17 | | Mean | | SD | |  |
| --- | --- | --- | --- | --- | --- | --- | --- | --- | --- | --- | --- | --- | --- | --- | --- | --- | --- | --- | --- | --- | --- | --- | --- | --- | --- | --- | --- | --- | --- | --- | --- |
| Phase | | M | | M | | M | | M | | M | | RS | | RS | | RS | | RS | | RS | | RC | | RC | | RC | | RC | | RC | |
| *Marinobacterium* | | 24.01 | | 40.00 | | 40.97 | | 34.99 | | 9.53 | | 61.34 | | 65.18 | | 0.00 | | 42.17 | | 36.57 | | 9.58 | | 8.62 | | 7.58 | | 8.59 | | 1.00 | |
| *Thalassolituus* | | 37.10 | | 39.67 | | 9.28 | | 28.68 | | 16.85 | | 0.00 | | 0.00 | | 0.00 | | 0.00 | | 0.00 | | 5.82 | | 4.46 | | 3.97 | | 4.75 | | 0.96 | |
| *Marinobacter* | | 16.00 | | 3.59 | | 7.59 | | 9.06 | | 6.33 | | 0.00 | | 0.00 | | 0.00 | | 0.00 | | 0.00 | | 3.74 | | 0.25 | | 0.02 | | 1.34 | | 2.08 | |
| *Desulfobacula* | | 4.74 | | 7.16 | | 7.14 | | 6.35 | | 1.39 | | 19.69 | | 16.35 | | 62.49 | | 32.84 | | 25.73 | | 12.47 | | 13.27 | | 20.41 | | 15.39 | | 4.37 | |
| *Alcanivorax* | | 0.71 | | 0.00 | | 11.26 | | 3.99 | | 6.31 | | 1.01 | | 0.18 | | 0.00 | | 0.40 | | 0.54 | | 5.03 | | 1.02 | | 29.51 | | 11.86 | | 15.42 | |
| *Alteromonas* | | 4.37 | | 0.00 | | 2.08 | | 2.15 | | 2.18 | | 0.99 | | 3.10 | | 0.00 | | 1.37 | | 1.59 | | 0.55 | | 0.58 | | 0.00 | | 0.38 | | 0.33 | |
| *Caldithrix* | | 0.00 | | 0.28 | | 0.55 | | 0.27 | | 0.27 | | 0.98 | | 0.83 | | 2.79 | | 1.53 | | 1.09 | | 1.41 | | 1.07 | | 0.46 | | 0.98 | | 0.48 | |
| *Pelobacter* | | 0.00 | | 0.00 | | 0.00 | | 0.00 | | 0.00 | | 0.00 | | 0.00 | | 0.00 | | 0.00 | | 0.00 | | 5.21 | | 2.22 | | 1.50 | | 2.98 | | 1.97 | |
| *Sulfurimonas* | | 0.00 | | 0.00 | | 0.00 | | 0.00 | | 0.00 | | 0.00 | | 0.00 | | 12.87 | | 4.29 | | 7.43 | | 0.00 | | 0.00 | | 0.00 | | 0.00 | | 0.00 | |

**Table S8:** Average relative percentage abundance of most abundant genera in OWCs with the mean and standard deviation of M, RS, and RC phases

**Table S9:** Overall average dissimilarity for different phases of seawater enrichments

|  | M | RS | RC |
| --- | --- | --- | --- |
| M |  | 85.1 | 91.54 |
| RS | 85.1 |  | 88.72 |
| RC | 91.54 | 88.72 |  |

**Table S10:** Overall average dissimilarity for different phases of oil enrichments

|  | M | RS | RC |
| --- | --- | --- | --- |
| M |  | 63.41 | 63.52 |
| RS | 63.41 |  | 65.5 |
| RC | 63.52 | 65.5 |  |

**Table S11:** Average relative percentage abundance of most abundant CCGs or CEGs in SWCs with the mean and standard deviation of M, RS, and RC phases

|  | **S1** | **S2** | **S3** | **Mean** | **SD** | **S4** | **S5** | **S6** | **Mean** | **SD** | **S7** | **S8** | **Mean** | **SD** |
| --- | --- | --- | --- | --- | --- | --- | --- | --- | --- | --- | --- | --- | --- | --- |
| **Phase** | **M** | **M** | **M** | **M** | **M** | **RS** | **RS** | **RS** | **RS** | **RS** | **RC** | **RC** | **RC** | **RC** |
| *Marinobacterium aestuarii* | 30.44 | 5.54 | 11.52 | 15.83 | 12.99 | 0.00 | 0.00 | 10.03 | 3.34 | 5.79 | 1.23 | 0.00 | 0.62 | 0.87 |
| *Marinobacter* sp. LQ44 | 9.67 | 0.00 | 18.71 | 9.46 | 9.36 | 0.00 | 0.00 | 0.00 | 0.00 | 0.00 | 0.00 | 0.00 | 0.00 | 0.00 |
| *Alteromonas macleodii* | 0.00 | 5.10 | 14.98 | 6.69 | 7.61 | 0.00 | 0.00 | 0.00 | 0.00 | 0.00 | 0.00 | 0.00 | 0.00 | 0.00 |
| *Desulfobacula toluolica* Tol2 | 6.21 | 1.71 | 7.49 | 5.13 | 3.04 | 8.52 | 17.54 | 21.27 | 15.78 | 6.56 | 0.74 | 2.46 | 1.60 | 1.21 |
| *Alteromonas mediterranea* U8 | 0.00 | 3.17 | 10.83 | 4.67 | 5.57 | 0.00 | 0.00 | 0.00 | 0.00 | 0.00 | 0.00 | 0.00 | 0.00 | 0.00 |
| *Cycloclasticus sp.* P1 | 2.32 | 8.94 | 2.69 | 4.65 | 3.72 | 0.00 | 0.00 | 0.00 | 0.00 | 0.00 | 0.00 | 0.00 | 0.00 | 0.00 |
| *Thalassolituus oleivorans* MIL-1 | 0.00 | 0.00 | 0.00 | 0.00 | 0.00 | 0.00 | 0.00 | 0.00 | 0.00 | 0.00 | 0.00 | 0.00 | 0.00 | 0.00 |
| *Thalassolituus oleivorans* | 0.00 | 6.34 | 3.55 | 3.30 | 3.18 | 0.00 | 0.00 | 0.00 | 0.00 | 0.00 | 0.00 | 0.00 | 0.00 | 0.00 |
| *Alcanivorax borkumensis* SK2 | 4.43 | 1.67 | 0.00 | 2.03 | 2.24 | 0.00 | 0.00 | 0.00 | 0.00 | 0.00 | 0.00 | 0.00 | 0.00 | 0.00 |
| *Desulfatibacillum alkenivorans* AK-01 | 2.62 | 0.00 | 1.37 | 1.33 | 1.31 | 2.34 | 0.00 | 6.89 | 3.08 | 3.50 | 1.29 | 37.70 | 19.50 | 25.75 |
| *Ca.* Puniceispirillum marinum IMCC1322 | 0.00 | 0.00 | 0.75 | 0.25 | 0.43 | 0.00 | 3.17 | 4.52 | 2.56 | 2.32 | 8.19 | 12.49 | 10.34 | 3.04 |
| *Pelobacter sp.* SFB93 | 0.40 | 0.00 | 0.00 | 0.13 | 0.23 | 0.00 | 0.00 | 0.00 | 0.00 | 0.00 | 0.00 | 0.00 | 0.00 | 0.00 |
| *Hyphomonas neptunium* ATCC 15444 | 0.00 | 0.00 | 0.00 | 0.00 | 0.00 | 0.00 | 0.00 | 0.00 | 0.00 | 0.00 | 4.03 | 26.53 | 15.28 | 15.91 |
| *Maricaulis maris* MCS10 | 0.00 | 0.00 | 0.00 | 0.00 | 0.00 | 0.00 | 1.18 | 0.00 | 0.39 | 0.68 | 5.77 | 2.87 | 4.32 | 2.05 |
| *Pseudomonas stutzeri* | 0.00 | 0.00 | 0.00 | 0.00 | 0.00 | 0.00 | 0.00 | 0.00 | 0.00 | 0.00 | 9.99 | 0.00 | 4.99 | 7.06 |
| *Sulfurimonas* | 0.00 | 0.00 | 0.00 | 0.00 | 0.00 | 80.55 | 56.27 | 35.09 | 57.31 | 22.75 | 0.79 | 0.00 | 0.39 | 0.56 |

**Table S12:** Average relative percentage abundance of most abundant CCGs or CEGs in OWCs with the mean and standard deviation of M, RS, and RC phases

|  | **S9** | **S10** | **S11** | **Mean** | **SD** | **S12** | **S13** | **S14** | **Mean** | **SD** | **S15** | **S16** | **S17** | **Mean** | **SD** |
| --- | --- | --- | --- | --- | --- | --- | --- | --- | --- | --- | --- | --- | --- | --- | --- |
| **Phase** | **M** | **M** | **M** | **M** | **M** | **RS** | **RS** | **RS** | **RS** | **RS** | **RC** | **RC** | **RC** | **RC** | **RC** |
| *Marinobacterium aestuarii* | 24.01 | 40.00 | 40.97 | 34.99 | 9.53 | 61.34 | 65.18 | 0.00 | 42.17 | 36.57 | 9.58 | 8.62 | 7.58 | 8.59 | 1.00 |
| *Marinobacter* sp*.* LQ44 | 14.83 | 1.97 | 1.69 | 6.16 | 7.51 | 0.00 | 0.00 | 0.00 | 0.00 | 0.00 | 1.10 | 0.00 | 0.00 | 0.37 | 0.64 |
| *Alteromonas macleodii* | 2.17 | 0.00 | 1.01 | 1.06 | 1.08 | 0.45 | 0.00 | 0.00 | 0.15 | 0.26 | 0.00 | 0.00 | 0.00 | 0.00 | 0.00 |
| *Desulfobacula toluolica* Tol2 | 4.74 | 7.16 | 7.14 | 6.35 | 1.39 | 19.69 | 16.35 | 62.49 | 32.84 | 25.73 | 12.47 | 13.27 | 20.41 | 15.39 | 4.37 |
| *Alteromonas mediterranea* U8 | 1.04 | 0.00 | 0.77 | 0.60 | 0.54 | 0.22 | 0.92 | 0.00 | 0.38 | 0.48 | 0.06 | 0.26 | 0.00 | 0.10 | 0.13 |
| *Cycloclasticus* sp. P1 | 0.00 | 0.00 | 0.00 | 0.00 | 0.00 | 0.00 | 0.00 | 0.00 | 0.00 | 0.00 | 0.00 | 0.00 | 0.00 | 0.00 | 0.00 |
| *Thalassolituus oleivorans* MIL-1 | 37.1 | 39.3 | 7.82 | 28.07 | 17.57 | 0.00 | 0.00 | 0.00 | 0.00 | 0.00 | 5.79 | 4.29 | 0.00 | 3.36 | 3.00 |
| *Thalassolituus oleivorans* | 0.02 | 0.36 | 1.45 | 0.61 | 0.75 | 0.00 | 0.00 | 0.00 | 0.00 | 0.00 | 0.03 | 0.17 | 3.97 | 1.39 | 2.24 |
| *Alcanivorax borkumensis* SK2 | 0.70 | 0.00 | 11.15 | 3.95 | 6.25 | 1.01 | 0.18 | 0.00 | 0.40 | 0.54 | 5.00 | 1.01 | 29.48 | 11.83 | 15.42 |
| *Desulfatibacillum alkenivorans* AK-01 | 0.00 | 0.00 | 0.90 | 0.30 | 0.52 | 0.27 | 0.00 | 0.00 | 0.09 | 0.16 | 0.00 | 0.00 | 0.00 | 0.00 | 0.00 |
| *Ca.* Puniceispirillum marinum IMCC1322 | 0.00 | 0.00 | 0.00 | 0.00 | 0.00 | 0.00 | 0.00 | 0.00 | 0.00 | 0.00 | 0.00 | 0.00 | 0.00 | 0.00 | 0.00 |
| *Pelobacter* sp. SFB93 | 0.00 | 0.00 | 0.00 | 0.00 | 0.00 | 0.00 | 0.00 | 0.00 | 0.00 | 0.00 | 5.21 | 2.22 | 1.48 | 2.97 | 1.98 |
| *Hyphomonas neptunium* ATCC 15444 | 0.00 | 0.00 | 0.00 | 0.00 | 0.00 | 0.00 | 0.00 | 0.00 | 0.00 | 0.00 | 0.00 | 0.00 | 0.00 | 0.00 | 0.00 |
| *Maricaulis maris* MCS10 | 0.00 | 0.00 | 0.00 | 0.00 | 0.00 | 0.00 | 0.00 | 0.00 | 0.00 | 0.00 | 0.00 | 0.00 | 0.00 | 0.00 | 0.00 |
| *Pseudomonas stutzeri* | 0.44 | 0.00 | 0.00 | 0.15 | 0.25 | 0.00 | 0.00 | 0.00 | 0.00 | 0.00 | 0.00 | 0.00 | 0.00 | 0.00 | 0.00 |
| *Sulfurimonas* | 0.00 | 0.00 | 0.00 | 0.00 | 0.00 | 0.00 | 0.00 | 12.87 | 4.29 | 7.43 | 0.00 | 0.00 | 0.00 | 0.00 | 0.00 |

**Table S13:** DESeq2 results showing significant fold changes of CCGs and CEGs between M vs. RS phases across SWCs and OWCs

|  | log2FoldChange | lfcSE | stat | *p*-value | *p*-adj |
| --- | --- | --- | --- | --- | --- |
| *Thalassolituus oleivorans* MIL-1 | 30.00 | 3.00 | 9.99 | 1.70E-23 | 8.95E-22 |
| *Sulfurimonas* | -29.82 | 3.00 | -9.93 | 3.08E-23 | 8.95E-22 |
| *Cycloclasticus* sp. P1 | 27.91 | 3.00 | 9.29 | 1.48E-20 | 2.86E-19 |
| *Rhodobacteraceae* | 27.41 | 3.00 | 9.13 | 6.97E-20 | 1.01E-18 |
| *Pelobacter carbinolicus* | 26.29 | 3.00 | 8.75 | 2.05E-18 | 2.38E-17 |
| *Candidatus Rickettsiella viridis* | 25.33 | 3.00 | 8.43 | 3.35E-17 | 3.24E-16 |
| *Alteromonadales* | 24.96 | 3.00 | 8.31 | 9.46E-17 | 7.84E-16 |
| *Ruegeria pomeroyi* | 24.22 | 3.00 | 8.06 | 7.59E-16 | 5.51E-15 |
| *Marinobacter* sp. LQ44 | 16.53 | 2.52 | 6.55 | 5.56E-11 | 3.59E-10 |
| *Marinobacter* sp. CP1 | 13.19 | 2.12 | 6.21 | 5.15E-10 | 2.99E-09 |
| *Marinobacter* | 11.94 | 2.04 | 5.85 | 4.81E-09 | 2.53E-08 |
| *Thalassolituus oleivorans* | 13.78 | 2.51 | 5.49 | 4.05E-08 | 1.96E-07 |
| *Marinobacter hydrocarbonoclasticus* | 13.08 | 2.43 | 5.38 | 7.45E-08 | 3.32E-07 |
| *Marinobacter hydrocarbonoclasticus* | 12.44 | 2.40 | 5.19 | 2.11E-07 | 8.76E-07 |
| *Marinobacter salinus* | 10.88 | 2.19 | 4.98 | 6.52E-07 | 2.52E-06 |
| *Marinobacter hydrocarbonoclasticus* | 9.74 | 2.73 | 3.57 | 0.000355 | 0.001287 |
| *Marinobacter* | 8.20 | 2.43 | 3.37 | 0.000747 | 0.002549 |
| *Alcanivorax* | 6.61 | 2.18 | 3.03 | 0.002425 | 0.007814 |
| *Pseudomonas sp.* LPH1 | 7.23 | 2.65 | 2.73 | 0.006319 | 0.018601 |
| *Celeribacter indicus* | 6.55 | 2.40 | 2.73 | 0.006414 | 0.018601 |
| *Oleiphilus messinensis* | 6.56 | 2.71 | 2.42 | 0.015338 | 0.040691 |
| *Alteromonas* *macleodii* | 6.99 | 2.89 | 2.42 | 0.015434 | 0.040691 |
| *Breoghania sp.* L-A4 | 6.51 | 3.00 | 2.17 | 0.030202 | 0.07616 |
| *Gammaproteobacteria* | 5.37 | 2.62 | 2.05 | 0.040351 | 0.097516 |

|  | log2FoldChange | lfcSE | stat | *p*-value | *p*-adj |
| --- | --- | --- | --- | --- | --- |
| *Candidatus Puniceispirillum marinum* | 30 | 2.78 | 10.79 | 3.7E-27 | 3.5E-25 |
| *Hyphomonas neptunium* | 30 | 2.97 | 10.10 | 5.3E-24 | 1.7E-22 |
| *Cycloclasticus* sp*.* P1 | 30 | 2.97 | 10.10 | 5.3E-24 | 1.7E-22 |
| *Thalassolituus oleivorans* | -29.68 | 2.98 | -9.96 | 2.2E-23 | 5.3E-22 |
| *Rhodobacteraceae* | 29.51 | 2.97 | 9.94 | 2.8E-23 | 5.4E-22 |
| *Maricaulis maris* | 28.60 | 2.97 | 9.63 | 5.9E-22 | 9.4E-21 |
| *Acidobacteria* | 27.31 | 2.87 | 9.52 | 1.8E-21 | 2.5E-20 |
| *Melioribacter roseus* | 27.90 | 2.97 | 9.39 | 5.7E-21 | 6.9E-20 |
| *Brevundimonas subvibrioides* | 27.40 | 2.97 | 9.23 | 2.8E-20 | 3E-19 |
| *Rhodobacteraceae* | 26.30 | 2.97 | 8.85 | 8.4E-19 | 8.1E-18 |
| *Confluentimicrobium* sp. EMB200-NS6 | 25.57 | 2.97 | 8.61 | 7.2E-18 | 6.3E-17 |
| *Brevefilum fermentans* | 25.49 | 2.97 | 8.58 | 9.4E-18 | 7.5E-17 |
| *Candidatus Rickettsiella viridis* | 24.63 | 2.97 | 8.29 | 1.1E-16 | 8.4E-16 |
| *Anaerolineaceae* | -20.31 | 2.98 | -6.81 | 9.9E-12 | 6.8E-11 |
| *Anaerolineaceae* | 9.11 | 2.97 | 3.07 | 0.00214 | 0.01361 |
| *Ruegeria pomeroyi* | 8.94 | 2.93 | 3.05 | 0.00227 | 0.01361 |
| Ignavibacteriales | 8.63 | 2.97 | 2.91 | 0.00365 | 0.02061 |
| *Hyphomonas* | 5.23 | 1.88 | 2.78 | 0.00543 | 0.02897 |
| *Rhodobacteraceae* | 8.11 | 2.97 | 2.73 | 0.00635 | 0.03188 |
| *Pelobacter carbinolicus* | 8.05 | 2.96 | 2.71 | 0.00664 | 0.03188 |
| *Thalassococcus sp.* SH-1 | 7.58 | 2.90 | 2.61 | 0.00895 | 0.04091 |
| *Desulfatibacillum alkenivorans* | 6.93 | 2.70 | 2.56 | 0.01038 | 0.04531 |
| *Sediminispirochaeta smaragdinae* | 6.33 | 2.49 | 2.55 | 0.01088 | 0.0454 |
| *Sulfurimonas* | 7.28 | 2.89 | 2.52 | 0.01178 | 0.04712 |
| *Phyllobacterium zundukense* | 7.37 | 2.97 | 2.48 | 0.01326 | 0.04992 |
| *Candidatus* Izimaplasma sp. HR1 | 7.15 | 2.90 | 2.47 | 0.01352 | 0.04992 |
| *Hyphomonas sp. Mor2* | 5.31 | 2.27 | 2.34 | 0.01915 | 0.06811 |
| *Pseudomonas* | 6.85 | 2.98 | 2.30 | 0.02144 | 0.0735 |
| Proteobacteria | 6.55 | 2.98 | 2.20 | 0.02795 | 0.09251 |
| *Pseudomonas stutzeri* | 5.94 | 2.99 | 1.99 | 0.04675 | 0.14959 |

**Table S14:** DESeq2 results showing significant fold changes of CCGs and CEGs between SWCs vs. OWCs across different phases

**Table S15:** DESeq2 results showing significant fold changes of CCGs and CEGs between RC vs. RS phases across SWCs and OWCs

|  | log2FoldChange | lfcSE | stat | *p*-value | *p*-adj |
| --- | --- | --- | --- | --- | --- |
| *Hyphomonas neptunium* ATCC 1544 | 30 | 3.01 | 9.95 | 2.49E-23 | 3.41E-22 |
| *Brevundimonas subvibrioides* | 30 | 3.01 | 9.95 | 2.49E-23 | 3.41E-22 |
| *Rhodobacteraceae* | 30 | 3.01 | 9.95 | 2.50E-23 | 3.41E-22 |
| *Thalassolituus oleivorans* MIL-1 | 28.03 | 3.02 | 9.30 | 1.43E-20 | 1.46E-19 |
| *Pelobacter sp.* SFB93 | 27.46 | 3.02 | 9.11 | 8.34E-20 | 6.84E-19 |
| *Candidatus Izimaplasma sp.* HR1 | -26.87 | 3.04 | -8.85 | 8.98E-19 | 6.14E-18 |
| *Acidobacteria* | -25.07 | 3.04 | -8.25 | 1.54E-16 | 9.03E-16 |
| Alphaproteobacteria | 8.30 | 3.02 | 2.75 | 0.00594 | 0.03046 |
| *Pseudomonas* | 7.66 | 3.02 | 2.53 | 0.01125 | 0.05125 |
| Proteobacteria | 7.36 | 3.02 | 2.43 | 0.01493 | 0.0612 |
| *Pseudomonas stutzeri* | 6.75 | 3.03 | 2.23 | 0.02591 | 0.09659 |
| *Maricaulis maris* MCS10 | 5.92 | 2.90 | 2.04 | 0.0412 | 0.13596 |
| *Gammaproteobacteria* | 5.38 | 2.66 | 2.02 | 0.04311 | 0.13596 |

**Table S16:** Summary of clustsig results showing *p*-values

|  | [,1] | [,2] | [,3] |
| --- | --- | --- | --- |
| [1,] | -12 | -13 | 1 |
| [2,] | -15 | -16 | 1 |
| [3,] | -9 | -10 | NA |
| [4,] | -4 | -5 | 1 |
| [5,] | -17 | 2 | 0.029029 |
| [6,] | -11 | 3 | 0.238238 |
| [7,] | -14 | 1 | 0.017017 |
| [8,] | -1 | -3 | NA |
| [9,] | -6 | -8 | NA |
| [10,] | -2 | 8 | 0.207207 |
| [11,] | -7 | 9 | 0.108108 |
| [12,] | 5 | 7 | 0.001001 |
| [13,] | 6 | 10 | 0 |
| [14,] | 4 | 11 | 0.003003 |
| [15,] | 12 | 13 | 0 |
| [16,] | 14 | 15 | 0 |

**Table S17:** Significant clusters observed from clustsig results. Significant clusters are marked with similar colours in Figure 3 of the manuscript.

| Cluster number | Samples | Samples | Samples |
| --- | --- | --- | --- |
| $significantclusters[[1]] | S4 | S5 |  |
| $significantclusters[[2]] | S7 | S6 | S8 |
| $significantclusters[[3]] | S17 |  |  |
| $significantclusters[[4]] | S15 | S16 |  |
| $significantclusters[[5]] | S14 |  |  |
| $significantclusters[[6]] | S12 | S13 |  |
| $significantclusters[[7]] | S11 | S9 | S10 |
| $significantclusters[[8]] | S2 | S1 | S3 |

**Table S18:** Summary of pvclust results where the #edge corresponds to the edge number mentioned in Figure S6

| #edge | au | bp | se.au | se.bp | v | c | pchi |
| --- | --- | --- | --- | --- | --- | --- | --- |
| 1 | 0.999 | 0.24 | 0 | 0.005 | -1.18 | 1.886 | 0.97 |
| 2 | 1 | 0.269 | 0 | 0.005 | -1.559 | 2.177 | 0.888 |
| 3 | 0.999 | 0.303 | 0 | 0.005 | -1.353 | 1.87 | 0.87 |
| 4 | 1 | 0.139 | 0 | 0.004 | -1.269 | 2.354 | 0.81 |
| 5 | 0.995 | 0.208 | 0.001 | 0.004 | -0.886 | 1.7 | 0.918 |
| 6 | 1 | 0.228 | 0 | 0.005 | -1.385 | 2.132 | 0.078 |
| 7 | 1 | 0.2 | 0 | 0.004 | -1.29 | 2.133 | 0.74 |
| 8 | 0.999 | 0.235 | 0 | 0.005 | -1.21 | 1.932 | 0.969 |
| 9 | 0.999 | 0.098 | 0 | 0.003 | -0.985 | 2.279 | 0.896 |
| 10 | 1 | 0.164 | 0 | 0.004 | -1.465 | 2.445 | 0.166 |
| 11 | 1 | 0.133 | 0 | 0.004 | -1.238 | 2.35 | 0.828 |
| 12 | 1 | 0.178 | 0 | 0.004 | -1.298 | 2.219 | 0.804 |
| 13 | 1 | 0.236 | 0 | 0.005 | -1.519 | 2.24 | 0.702 |
| 14 | 1 | 0.191 | 0 | 0.004 | -1.212 | 2.087 | 0.335 |
| 15 | 1 | 0.148 | 0 | 0.004 | -1.148 | 2.195 | 0.813 |
| 16 | 1 | 0.104 | 0 | 0.003 | -1.167 | 2.424 | 0.882 |
| 17 | 0.999 | 0.167 | 0 | 0.004 | -1.124 | 2.091 | 0.709 |
| 18 | 1 | 0.176 | 0 | 0.004 | -1.367 | 2.296 | 0.329 |
| 19 | 0.996 | 0.051 | 0.002 | 0.002 | -0.496 | 2.135 | 0.85 |
| 20 | 0.998 | 0.061 | 0.001 | 0.003 | -0.69 | 2.24 | 0.795 |
| 21 | 0.999 | 0.082 | 0.001 | 0.003 | -0.864 | 2.254 | 0.314 |
| 22 | 0.999 | 0.084 | 0.001 | 0.003 | -0.799 | 2.176 | 0.503 |
| 23 | 1 | 0.023 | 0 | 0.002 | -0.857 | 2.858 | 0.914 |
| 24 | 1 | 0.079 | 0 | 0.003 | -1.1 | 2.51 | 0.602 |
| 25 | 1 | 0.025 | 0 | 0.002 | -0.788 | 2.753 | 0.783 |
| 26 | 0.999 | 0.028 | 0.001 | 0.002 | -0.569 | 2.485 | 0.442 |
| 27 | 1 | 0.037 | 0 | 0.002 | -0.896 | 2.679 | 0.545 |
| 28 | 1 | 0.009 | 0.001 | 0.001 | -0.499 | 2.852 | 0.574 |
| 29 | 1 | 0.034 | 0 | 0.002 | -0.786 | 2.618 | 0.425 |
| 30 | 0.999 | 0.337 | 0 | 0.005 | -1.302 | 1.723 | 0.955 |

**Table S19:** Comparative composition of natural seawater and synthetic seawater (Instant Ocean) used in this study

| Ion | Natural Seawater  (g/kg) | Instant Ocean  (g/kg) |
| --- | --- | --- |
| Sodium | 10.781 | 10.780 |
| Potassium | 0.399 | 0.420 |
| Magnesium | 1.284 | 1.320 |
| Calcium | 0.4119 | 0.400 |
| Strontium | 0.00794 | 0.00880 |
| Chloride | 19.353 | 19.290 |
| Sulphate | 2.712 | 2.660 |
| Bicarbonate | 0.126 | 0.200 |
| Bromide | 0.0673 | 0.056 |
| Boric acid | 0.0257 | - |
| Fluoride | 0.00130 | 0.001 |

**Figures**


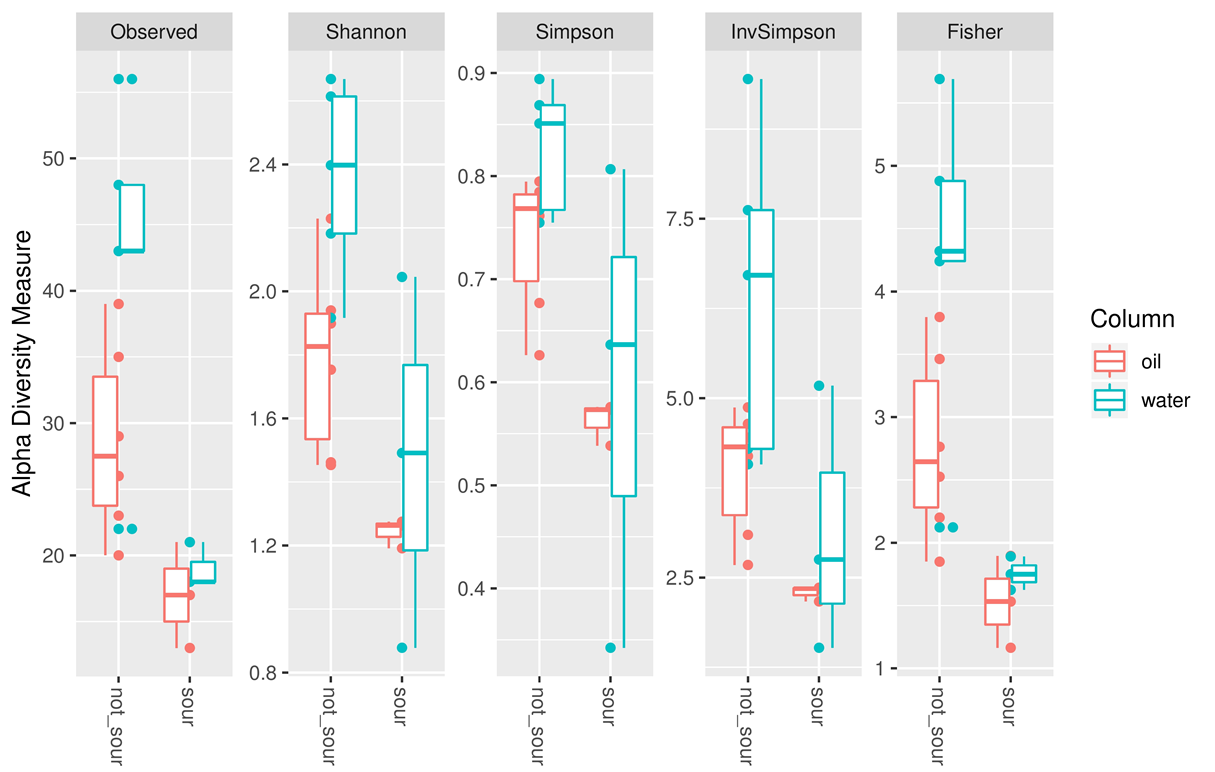


**Figure S1:** Box plot of alpha diversity indices observed across sour and non-sour phases of seawater and oil enrichment.

**Figure S2:** Average relative abundance of Bacteroidetes across different phases of oil enrichment.


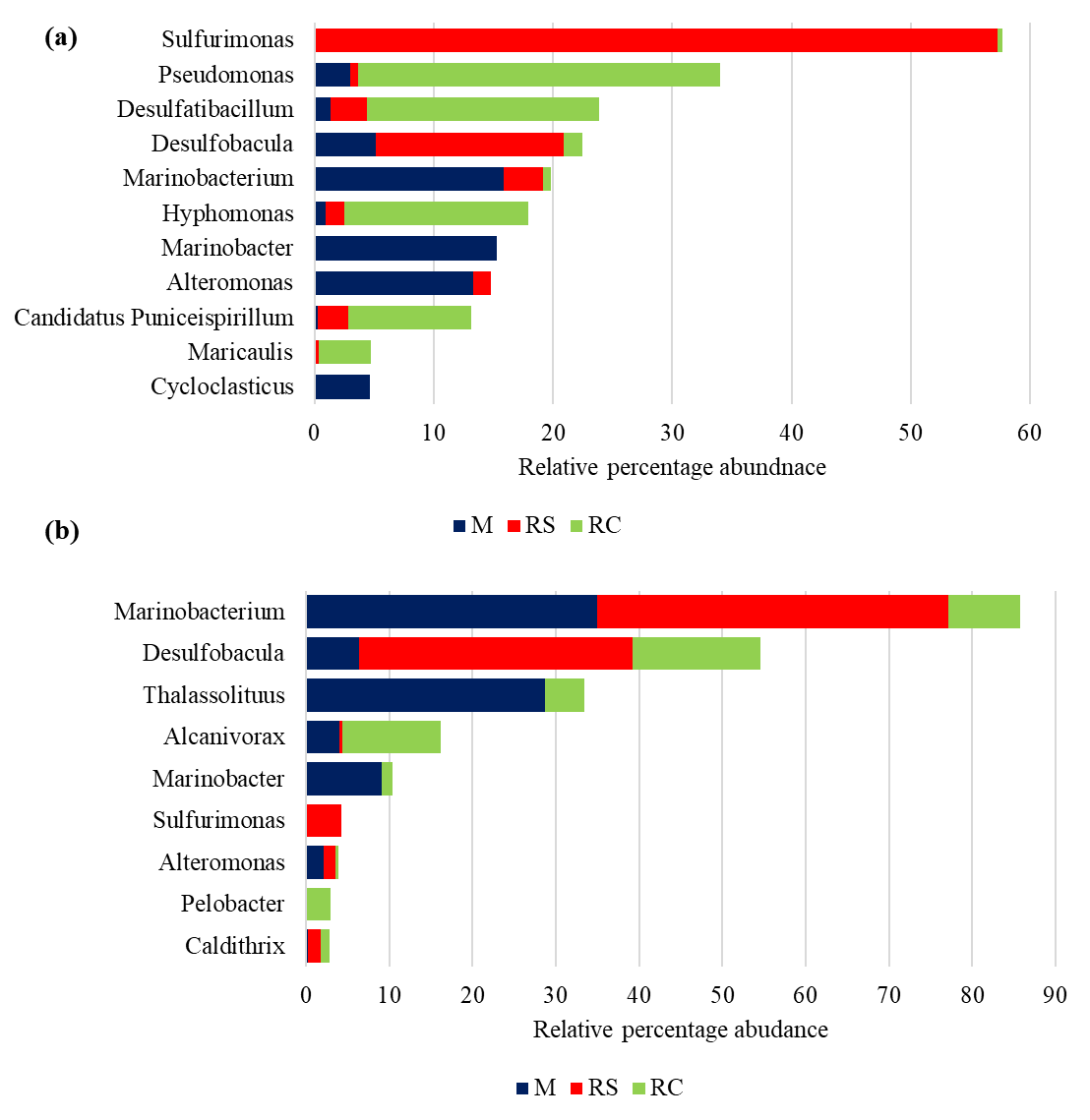


**(B)**

**(A)**

**Figure S3:** Distribution of the five most abundant genera observed across different phases in **(A)** SWCs and **(B)** OWCs. Average abundances of each genus across different phases are plotted.

**Figure S4:** Plot showing relative abundance of *Desulfobacula toluolica* Tol2 across M and RS phases in different columns. Columns 1, 2, and 3 are seawater-wetted columns and Columns 4, 5, and 6 are oil-wetted columns


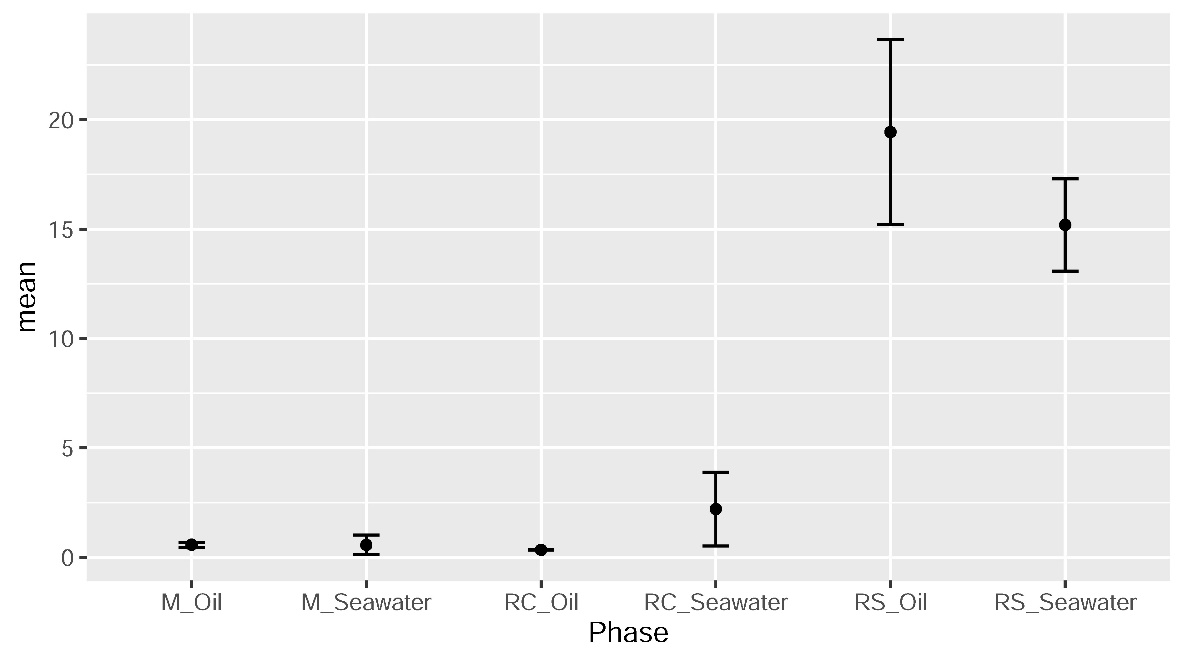


**Figure S5:** Plot showing mean and standard deviation of sulfide production (in mg L^-1^) across different phases of SWCs and OWCs


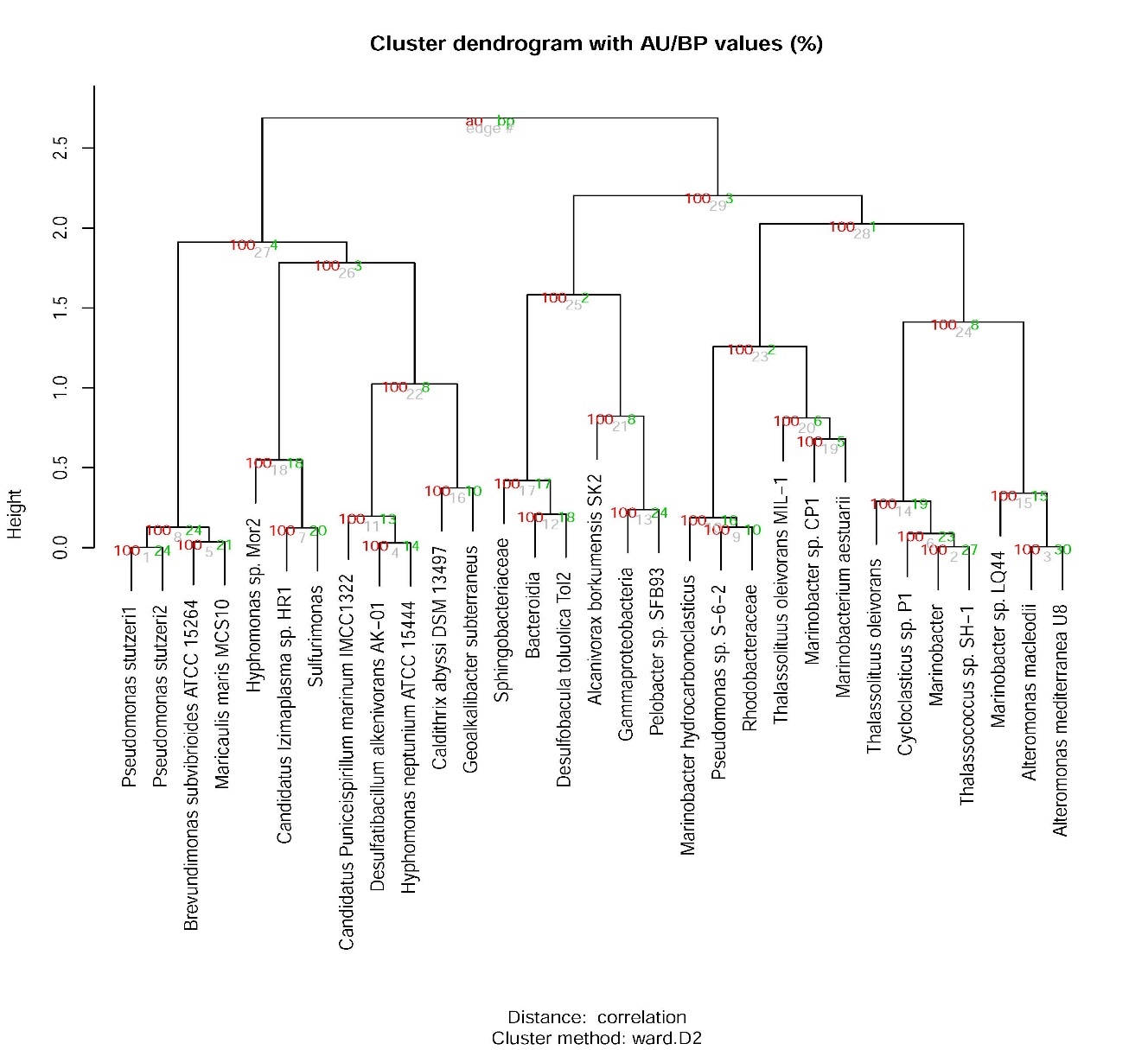


**Figure S6:** Dendogram showing au (approximately unbiased) *p*-values and bp (bootstrap probability) values for different clusters of the most abundant taxa. Clusters are considered significant for AU > 95 indicating 95% probability of not being a random cluster.
